# Yellow Mosaic Disease (YMD) of Mungbean (*Vigna radiata* (L.) Wilczek): Current Status and Management Opportunities

**DOI:** 10.3389/fpls.2020.00918

**Published:** 2020-06-24

**Authors:** Gyan P. Mishra, Harsh K. Dikshit, Ramesh S. V., Kuldeep Tripathi, Ranjeet R. Kumar, Muraleedhar Aski, Akanksha Singh, Anirban Roy, Priti, Nikki Kumari, Uttarayan Dasgupta, Atul Kumar, Shelly Praveen, Ramakrishnan M. Nair

**Affiliations:** ^1^Division of Genetics, ICAR-Indian Agricultural Research Institute, New Delhi, India; ^2^Division of Physiology, Biochemistry and PHT, ICAR-Central Plantation, Kasaragod, India; ^3^Germplasm Evaluation Division, ICAR-National Bureau of Plant Genetic Resources, New Delhi, India; ^4^Division of Biochemistry, ICAR-Indian Agricultural Research Institute, New Delhi, India; ^5^Division of Plant Pathology, ICAR-Indian Agricultural Research Institute, New Delhi, India; ^6^Division of Seed Science and Technology, ICAR-Indian Agricultural Research Institute, New Delhi, India; ^7^World Vegetable Center, South Asia, ICRISAT Campus, Patancheru, Hyderabad, India

**Keywords:** begomovirus, gene editing, greengram, pathogen derived resistance, translational genomics, vector management

## Abstract

Globally, yellow mosaic disease (YMD) remains a major constraint of mungbean production, and management of this deadly disease is still the biggest challenge. Thus, finding ways to manage YMD including development of varieties possessing resistance against mungbean yellow mosaic virus (MYMV) and mungbean yellow mosaic India virus (MYMIV) is a research priority for mungbean crop. Characterization of YMD resistance using various advanced molecular and biochemical approaches during plant–virus interactions has unfolded a comprehensive network of pathogen survival, disease severity, and the response of plants to pathogen attack, including mechanisms of YMD resistance in mungbean. The biggest challenge in YMD management is the effective utilization of an array of information gained so far, in an integrated manner for the development of genotypes having durable resistance against yellow mosaic virus (YMV) infection. In this backdrop, this review summarizes the role of various begomoviruses, its genomic components, and vector whiteflies, including cryptic species in the YMD expression. Also, information about the genetics of YMD in both mungbean and blackgram crops is comprehensively presented, as both the species are crossable, and same viral strains are also found affecting these crops. Also, implications of various management strategies including the use of resistance sources, the primary source of inoculums and vector management, wide-hybridization, mutation breeding, marker-assisted selection (MAS), and pathogen-derived resistance (PDR) are thoroughly discussed. Finally, the prospects of employing various powerful emerging tools like translational genomics, and gene editing using CRISPR/Cas9 are also highlighted to complete the YMD management perspective in mungbean.

## Introduction

Mungbean (*Vigna radiata* (L.) Wilczek) is indigenous to India or Indo-Burma region and is the third most important self-pollinated, short**-**duration grain legume crop after chickpea and pigeonpea. The central Asian region is believed to be the primary center of genetic diversity for mungbean (Kumar and Kumar, 2014). The genome size of mungbean is relatively small (579 Mb) and the 2n number of chromosomes is 22 (Parida et al., 1990; Kang et al., 2014). It is also known as greengram, greenbean, mashbean, goldengram, and greensoy (Markam et al., 2018). Mungbean is an important and cheap source of food protein across Asia, especially for the poor, thus plays an imperative role in the alleviation of protein malnutrition especially in the developing countries (Selvi et al., 2006). It contains a relatively high proportion of easily digestible good quality protein (24%) with low flatulence and is also rich in iron contents (40–70 ppm), making it an ultimate choice for balanced diets (Selvi et al., 2006; Vairam et al., 2016).

Besides seeds, its sprouts, which contain high vitamin C and folate are also very much relished in Asian cuisine (Nair et al., 2013); while its foliage can also be used as fodder, feed, and hay. *Rhizobium* and *Bradyrhizobium* bacteria which are present in the root-nodules of mungbean, fix the atmospheric nitrogen and thus improve the soil fertility, and benefit the succeeding crops. Mungbean is being cultivated across a wide range of latitudes (40 N or S) covering tropical and sub**-**tropical regions of the world and is suitably adapted to a range of cropping systems (http://avrdc.org/intl-mungbean-network/). Globally, mungbean is being grown in over 7.0 million ha area, yielding 3.5 million tons of grains mainly from Asia but spreading to other parts of the world (Nair et al., 2019). The major mungbean growing countries include India, China, Pakistan, Bangladesh, Sri Lanka, Thailand, Myanmar, Vietnam, Indonesia, Australia, and the Philippines (Alam et al., 2014b). Worldwide, India is the largest mungbean producer, yielding 2.17 million tons of grains from about 4.32 m ha area. However, the average productivity of mungbean in India is quite low (~502 kg/ha), even lower than most of the other pulse crops (Project Coordinators Report-2018).

In mungbean, yellow mosaic disease (YMD) caused by yellow mosaic viruses (YMVs) is of key importance especially in South and Southeast Asia. Besides mungbean, YMD also affect various leguminous crops including blackgram (*Vigna mungo*), mothbean (*Vigna aconitifolia*), Lima bean (*P. lunatus*), pigeonpea (*Cajanus cajan*), French bean (*Phaseolus vulgaris*), cowpea (*Vigna unguiculata*), Dolichos (*Lablab purpureus*), horsegram (*Macrotyloma uniflorum*), and soybean (*Glycine max*) (Ramesh et al., 2017b; Dikshit et al., 2020). The overall crop yield loss may range between 10 and 100%, depending on the mungbean genotype and stage of crop infection (Singh, 1980a; Marimuthu et al., 1981; Bashir et al., 2006).

**Figure 1:**
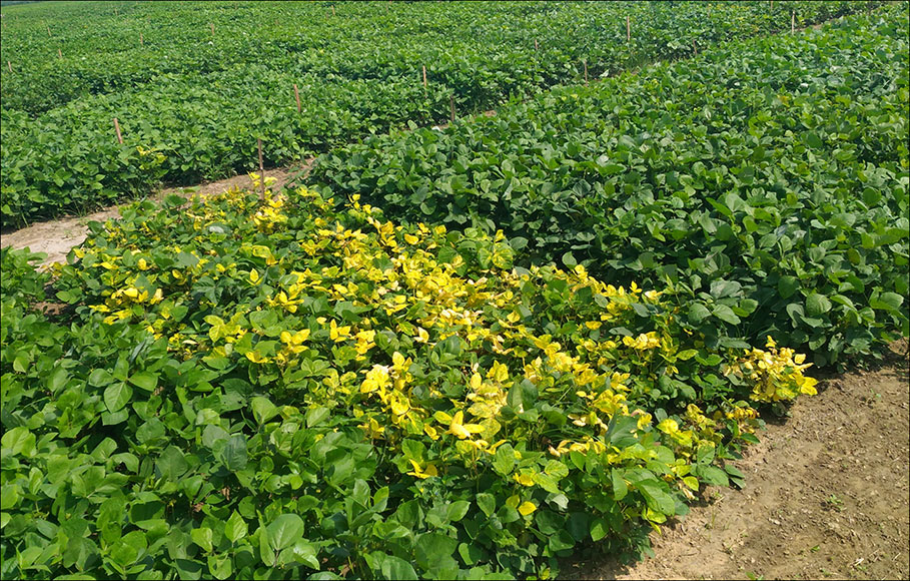
Field view of YMD susceptible (yellowing of the plants) and resistant expression (normal green plants) in various mungbean genotypes.

YMD spread to the mungbean crop through whitefly (*Bemisia tabaci* Gennadius)—an insect vector for YMVs (Selvi et al., 2006). Although, YMD has been reported throughout the world (except Australia); but its heavy incidence is mainly reported from countries like India, Bangladesh, and Pakistan (Pathak and Jhamaria, 2004; Biswas et al., 2008; Salam et al., 2011). The virus enters the phloem cells of the host through the whitefly proboscis and the viral aggregates appear in the host cell nuclei roughly two days before the symptom appearance (Thongmeearkom et al., 1981). The visible symptoms appear as scattered yellow-color spots on the young leaves which later turns into a yellow mosaic pattern and ultimately results in complete yellowing, drying and withering of leaves (**Figure 1**). The pods on the infected mungbean plant become smaller in size, yellowing of the leaves decreases the photosynthetic efficiency which ultimately manifested as severe yield penalty (Malathi and John, 2009).

In India, MYMV was first reported from the mungbean fields of Indian Agricultural Research Institute (IARI), New Delhi during 1950s (Nariani, 1960). In general, MYMV is the major isolate infecting mungbean crop in western and southern India, Thailand, and Indonesia; whereas, MYMIV isolate in central, eastern and northern India, Pakistan, Bangladesh, Nepal, and Vietnam (Malathi and John, 2009). With this background, this review systematically deals with the scientific developments about YMVs infecting mungbean, its vector and also various YMD management challenges including the prospective use of recent tools like—omics approaches and translational genomics, across the world.

## Begomovirus and YMD in *Vigna*

The family *Geminiviridae* comprised of nine genera, *viz*., *Becurtovirus*, *Begomovirus*, *Capulovirus*, *Curtovirus*, *Eragrovirus*, *Grablovirus*, *Mastrevirus*, *Topucovirus*, and *Turncurtovirus*, and the viruses are attributed to respective genus depending on its host, vector and genome arrangements (Varsani et al., 2017; Zerbini et al., 2017). The genus name *Begomovirus* was derived from the type member, *Bean Golden MOsaic virus* (BGMV), causing golden mosaic disease in beans. *Begomovirus* is the largest genus of a family *Geminiviridae* having twinned quasi-icosahedral particles (20 × 30 nm) encapsidating circular ss-DNA. These are mostly bipartite, with vector specificity and have specific amino acid sequences in its coat protein (Briddon and Markham, 2000). It comprises of nearly 322 species and more than 500 isolates, infecting various economically important dicot crops (Fauquet et al., 2008; Varsani et al., 2014; Varsani et al., 2017).

In pulses, depending on the viral nucleotide sequence identity, yellow mosaic disease (YMD) is caused by four distinct begomoviruses namely, (i) MYMV, (ii) MYMIV, (iii) dolichos yellow mosaic virus (DoYMV) and (iv) horsegram yellow mosaic virus (HgYMV); which are collectively known as yellow mosaic viruses (YMVs) (Qazi et al., 2007; Malathi and John, 2009; Naimuddin et al., 2016). The term ‘Legumoviruses’ has been used to refer the legume infecting bipartite begomoviruses (Briddon et al., 2010).

**Figure 2:**
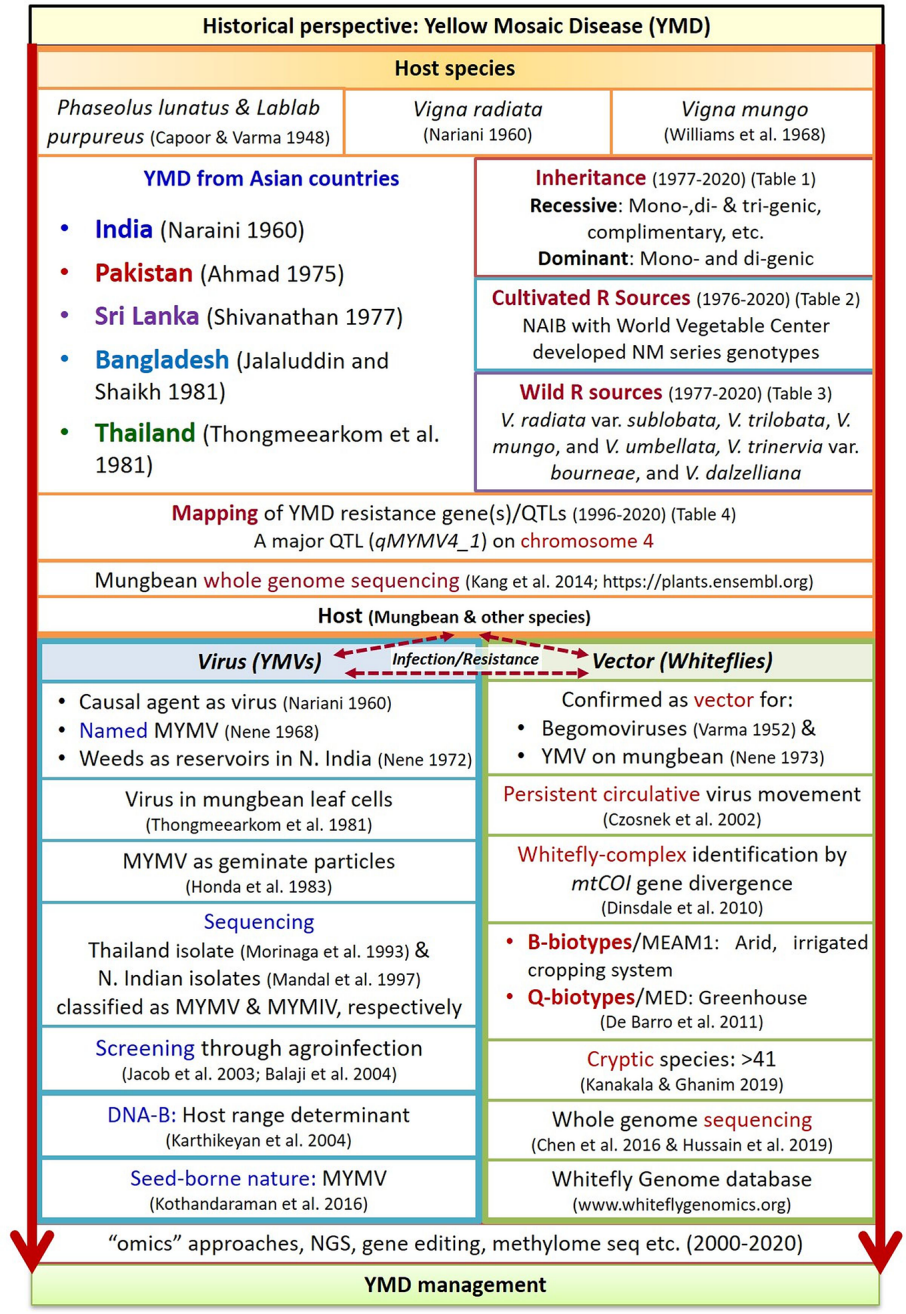
Historical sketch of YMD in mungbean crop (Derived from Capoor and Varma, 1948; Capoor and Varma, 1950; Varma, 1952; Nariani, 1960; Nene, 1968; Williams et al., 1968; Nene, 1972; Nene, 1973; Ahmad, 1975; Shivanathan, 1977; Jalaluddin and Shaikh, 1981; Thongmeearkom et al., 1981; Honda et al., 1983; Morinaga et al., 1993; Mandal et al., 1997; Czosnek et al., 2002; Jacob et al., 2003; Balaji et al., 2004; Karthikeyan et al., 2004; Dinsdale et al., 2010; De Barro et al., 2011; Kang et al., 2014; Chen et al., 2016; Kothandaraman et al., 2016; Hussain et al., 2019; Kanakala and Ghanim, 2019). Where, R, resistance; YMVs, yellow mosaic viruses; NGS, next generation sequencing.

MYMV particles were first observed and purified in the leaf cells of mungbean by Thongmeearkom et al. (1981) and Honda et al. (1983), respectively. The genome of Thailand isolates of MYMV (Morinaga et al., 1993) and the isolate from North India (Mandal et al., 1997) was found sharing <89% similarity (Fauquet et al., 2008), hence considered as a distinct species, and later was named as MYMIV. The detailed historical perspectives of YMD in mungbean are presented in chronological order in **Figure 2**.

## Role of various DNA components in the YMD Expression and Molecular Characterization of Begomoviruses

Initially, all the begomoviruses were considered to be monopartite and DNA-B was believed to be generated as a satellite, which later got established as an integral part of the genome. The DNA-A and DNA-B of bipartite begomoviruses were supposed to be unique and diversification in these is due to the component exchange during evolution (Briddon et al., 2010). The viral sense strand of DNA-A encodes the coat protein (CP, ~29.7 kDa) and movement or pre-coat protein (~12.8 kDa) from AV1 and AV2 genes, respectively. The MYMIV-AV2 protein was also reported modulating the functions of *Rep* protein by affecting the ratio between open circular and supercoiled DNA forms (Rouhibakhsh et al., 2011).

**Figure 3:**
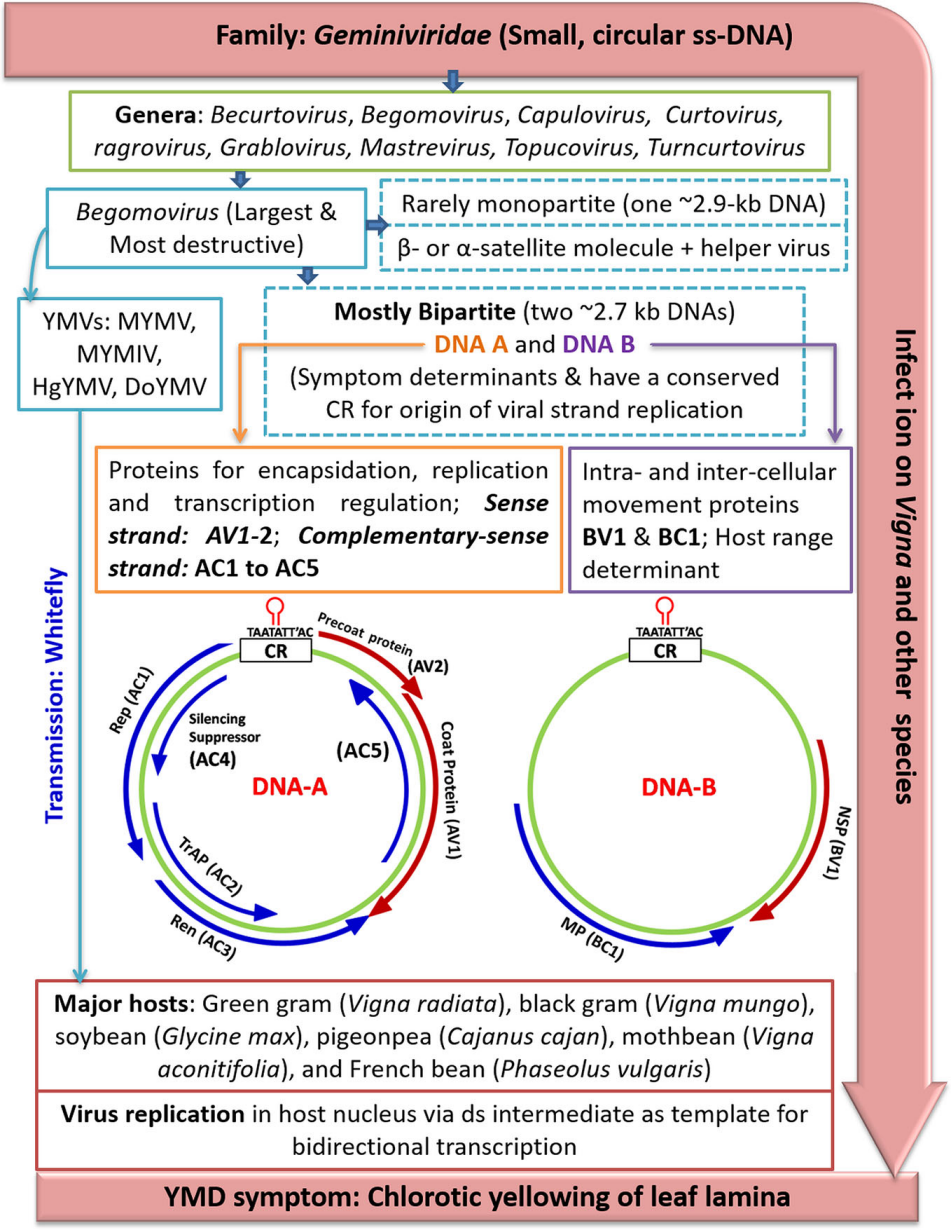
An outline of YMD development in mungbean. Where, YMD (yellow mosaic disease), MYMV (mungbean yellow mosaic virus), MYMIV (mungbean yellow mosaic India virus), HgYMV (horsegram yellow mosaic virus), DoYMV (dolichos yellow mosaic virus), AV2 (precoat protein), CP/AV1 (coat protein), Rep/AC1 (replication protein), TrAP/AC2 (transcriptional activator protein), REn/AC3 (rep enhancer protein), AC4 (silencing suppressor), CR (common region). The maps of YMV genomic DNA-A and DNA-B components are derived from Kumar et al. (2017b) and Shivaprasad et al. (2005) in which the ORF (open reading frames) are presented as bar arrows with the head representing 3′-terminus.

The viral complementary sense strand encodes four proteins namely, replication-associated protein (*Rep*, ~40.2 kDa; ORF AC1), replication enhancer protein (*REn*, ~15.6 kDa; ORF AC3) and transcription activator protein (*TrAP*, ~19.6 kDa; ORF AC2). The AC4 (~12.0 kDa) is believed to regulate symptom expression; whereas, AC5 which is located downstream of AC3 (in antisense orientation of DNA-A) codes for a pathogenicity determinant in MYMIV, suppressing only sense RNA-induced gene silencing (Li et al., 2015) (**Figure 3**).

The DNA-B harbors two genes viz., BV1 (in viral sense strand) and BC1 (in complementary sense strand) encoding nuclear shuttle protein (NSP; ~33.1 kDa) and movement protein (MP; ~29.6 kDa), respectively. The MP regulates the cell to cell movement of viruses *via* plasmodesmata, while NSP helps in the movement of viral DNA between the host cell nucleus and cytoplasm and also their long-distance movement through host vascular system (Hanley-Bowdoin et al., 1999). Since the BV1 and BC1 are absent in monopartite begomoviruses, the function of NSP is found played by CP (AV1) gene (Polston et al., 1997). Also, the mungbean plant proteins are reported influenced by the plant–virus interaction and are simultaneously used by the viruses for its growth, multiplication, and cell-to-cell movement (Cayalvizhi et al., 2015).

A 200 bp region common to both DNA-A and DNA-B of bipartite begomoviruses is known as the common region (CR). The intergenic region in begomoviruses possesses an origin of replication (ori), a highly conserved stem-loop or hairpin structure having a nonanucleotide motif (TAATATT↓AC) and ‘iterons’ or direct repeat motifs of 5–7 nucleotide length (Hanley-Bowdoin et al., 1999; Pant et al., 2001).

Iterons function by recognizing the *Rep* proteins which nick the nonanucleotide motif and start the rolling circle DNA replication (Argüello-Astorga et al., 1994). Both DNA-A and DNA-B contains very similar iteron sequences which ensure that the DNA-A encoded Rep can initiate replication of both components (Shafiq et al., 2010). Highly specific Rep-Iteron interaction prevents the interaction between distinct begomovirus species (Chatterji et al., 2000) and thereby maintains the bipartite genome integrity. The iteron sequence (GGTGT) of MYMV, MYMIV, and DoYMV are similar, whereas HgYMV has a different sequence (GGTAT), thus are unable to readily exchange its components with other legume yellow mosaic viruses (LYMVs). Moreover, due to recombination, there is a replacement between *ori* of DNA-B with that of DNA-A, resulting in component capture between distinct species as reported for the HgYMV-like DNA-B sequence containing iteron motifs GGTGT (Girish and Usha, 2005).

### Molecular Characterization of YMVs

YMVs are mostly characterized either by complete sequencing or by sequencing of various DNA-A and DNA-B components. Molecular characterization of YMVs infecting mungbean in Bangladesh and Pakistan revealed 97 and 94% sequence similarity for the CP and NSP-genes of MYMIV, respectively (Hussain et al., 2004; Islam et al., 2012). Similarly, sequence-based phylogenetic analysis of legume-infecting begomoviruses from Indonesia and Vietnam has identified Indonesian isolates as MYMIV strain-A, while Vietnam isolates as MYMV strain-B (Tsai et al., 2013). Furthermore, sequencing of 44 components (23 DNA-A, 19 DNA-B, and 2 betasatellites) of various LYMVs occurring across Pakistan revealed the presence of showed MYMIV with two distinct types (Ilyas et al., 2010). Molecular analysis of a begomovirus infecting *V. mungo* var. *Silvestris*, revealed it to be a strain of MYMIV and is designated as MYMIV-VSKN (Naimuddin et al., 2011b). CP gene characterization revealed considerable genetic variability in the MYMV-Tamil Nadu isolates of blackgram, cowpea and mungbean samples (Maheshwari et al., 2014). Molecular studies identified MYMIV isolates causing YMD in blackgram collected from Andhra Pradesh (India); whereas, MYMV isolate was found in the neighboring state of Tamil Nadu (Reddy et al., 2015). Recently, a new isolate (Mg-mungbean-1) of MYMIV having a recombinant DNA-B component was identified from Meghalaya (India). The DNA-A based phylogenetic tree also confirmed this novel isolate as a MYMIV (Banerjee et al., 2018).

### Role of DNA Components in Infection by YMVs

Less durability of resistance of a legume genotype against begomoviruses may be due to recombination and component exchanges occurring in the viruses. However, no comprehensive evidence indicating the interaction of virus infecting various legume species exist, which means legume infecting begomoviruses are evolving independently of those infecting other plant families (Qazi et al., 2007). The DNA-B of an HgYMV isolate showed very high sequence similarity (96%) with that of soybean MYMV isolate, while it was only 70–73% with MYMV and MYMIV DNA-B which is speculated to be due to the component exchange and appears as host range expansion adaptation mechanism (Qazi et al., 2007).

Kumar et al. (2017a) through agroinoculation of dimeric infectious clones (having both DNA-A and DNA-B of MYMIV) have confirmed the pathogenicity of cowpea strain of MYMIV in cowpea and mungbean. Further sequence analysis has confirmed it as MYMIV isolate harboring a distinct DNA-B component playing a key role in symptom expression. Interestingly, viral clones were infectious to various crops (viz. cowpea, mungbean, blackgram, and French bean), but wild-type isolates are transmissible *via* whiteflies to only cowpea and not to blackgram or mungbean, suggesting the role of insect vector determining the natural host range (John et al., 2008). Ilyas et al. (2010) using sequence information of LYMVs revealed that either recombination with nonlegume viruses or interactions with betasatellites of begomoviruses is the reason for the emergence of more virulent variants affecting various legumes.

The comparison of blackgram isolate of MYMV (IMYMV-Bg) showed sequence divergence for the common region (CR) between DNA-A and DNA-B, while overexpression of IMYMV-Bg *Rep* protein in *E. coli* showed its specific binding to the CR-sequences. In addition, ATP-upregulated cleavage and ATP-mediated conformational change of *Rep* was also recorded (Pant et al., 2001). The agroinoculation of partial dimers of KA27 and KA22 DNA-Bs with DNA-A in blackgram and mungbean, established DNA-B of MYMV-Vig as a vital host-range determinant (Balaji et al., 2004). The swapping of the KA27 DNA-B component with the KA22 DNA-B nuclear shuttle protein (NSP) gene in MYMV-Vig has resulted in mild-yellow symptoms, suggesting NSP as major symptom determinant (Mahajan et al., 2011). The cloned DNA-A and five different DNA-Bs of MYMV-Vig when agroinoculated with mixed cultures of *Agrobacterium* showed co-infection ability of all DNA-B components to *V. mungo* (Karthikeyan et al., 2004). Thus, the co-existence of multiple DNA-B components of MYMV-Vig appears helping its host range expansion, while additional DNA-B components may help in infecting *V. radiata* and *V. aconitifolia* (Karthikeyan et al., 2004). Hence, it looks obligatory to find a more precise role being played by different DNA components of various YMVs affecting diverse *Vigna* species. A comprehensive list of primers amplifying different MYMV components as reported by different researchers is presented in **Table S1**.

## Genetics of YMD Resistance IN *Vigna*

Most commonly exploited measure for YMD management in mungbean is the development and use of resistant varieties. However, the nature of the gene(s) controlling the YMD resistance in *Vigna* is reported varying in different genetic backgrounds and gene actions differ from a single dominant gene (Markam et al., 2018) to the recessive gene inheritance (Khattak et al., 2000; Dhole and Reddy, 2012) (**Table 1**). YMD tolerance and resistance were found regulated by one and two recessive genes, respectively in different cross combinations, while pairs of genes having dominant and recessive epistasis were also found governing resistance in interspecific crosses (Singh and Sharma, 1983).

**Table 1:** Genetics of MYMV resistance in mungbean, blackgram, and interspecific crosses.

S. No.	Generation/s	Resistant, Susceptible parent/Cross	Genetics of resistance	Reference
**Mungbean**				
**1**.	F_2_ and F_3_	6601, NM92 (R); VC1560D, VC3902A, Berken (S); KMG189 × VBN(Gg)2	Monogenic recessive	Khan et al., 2007; Reddy, 2009; Dhole and Reddy, 2013; Sudha et al., 2013b
**2**.	F_2_	–	Complementary recessive genes	Shukla and Pandya, 1985; Alam et al., 2014a
**3**.	F_2_	–	Dominant and complementary recessive genes	Sandhu et al., 1985
**4**.	F_2_	SML668 (S) × Mash114 (R)	Monogenic dominant gene	Lekhi et al., 2018
**5**.	–	–	Trigenic recessive	Mishra and Asthana, 1996
**6**.	F_1_, F_2_ and F_3_	NM92, ML-5, Var.6601 (R); VC2272, Pusa Baisakhi, VC1560D, VC3902A, Berken, Emerald (S)	Modifying genes	Khattak et al., 2000
**7**.	F_1_, F_2,_ BC_1_, and BC_2_	ML818, Satya (R); SML32, Koppergoan (S)	Digenic recessive	Ammavasai et al., 2004; Dhole and Reddy, 2012; Singh et al., 2013
**8**.	F_2_	HUM12, SML1455, AKM9904 (S); Pusa0672, ML1464 (R)	Digenic recessive	Bhanu et al. (2019)
**9**.	F_1_ and F_2_	SML1815, IPM19, Pusa Vishal, Pusa9072, Malviya Jyoti, HUM12, CO6, MH934, MH421, COGG11**-**02, VGG10**-**008	Trigenic (02 dominant + 01 recessive)	Markam et al., 2018
**10**.	F_2_	VBN(Gg)2 × SML1815, VBN(Gg)3 × SML1815, VBN (Gg)3 × MH421	Digenic dominant	Mahalingam et al., 2018
**Blackgram**				
**11**.	F_2_	–	Digenic recessive	Singh, 1980a; Verma and Singh, 1986
**12**.	F_2_	–	Monogenic dominant	Kaushal and Singh, 1988; Gupta et al., 2005
**13**.	F_2_ and back-cross	Blackgram crosses	Monogenic recessive	Pal et al., 1991
**14**.	F_2_	KMG189 (R); VBN(Gg)2 (S)	Monogenic recessive	Basak et al., 2005; Sai et al., 2017
**15**.	F_2_	Co5 × VBN(Bg)4, Co5 × VBG66	Digenic and Trigenic dominant	Murugan and Nadarajan, 2012
**16**.	F_2_	–	Digenic dominant	Durga Prasad et al., 2015
**17**.	F_1_, F_2,_ BC_1_and BC_2_	MDU1 × Mash-114, MDU1 × VBN (Bg)6, MDU × PU31, MDU1 × Uttara, LPG752 × Mash-114, LPG752 × VBN(Bg)6, CO6 × VBN(Bg)6	Digenic dominant with epistasis	Thamodhran et al., 2016
**Inter-specific cross**				
**18**.	–	*V. radiata* × *V. radiata* var. *Sublobata*	Dominant and recessive Epistasis	Singh and Sharma, 1983
**19**.	–	Wide cross of blackgram	Digenic recessive	Dwivedi and Singh, 1985
**20**.	F_2_and back-cross	Mungbean × blackgram; Mungbean × *V. sublobata*	Digenic recessive	Pal et al., 1991
**21**.	F_2_ andF_3_	TNAU RED × VRM(Gg)1	Monogenic recessive	Sudha et al., 2013b
**22**.	RIL	*V. radiata × V. umbellata*	Major QTL	Mathivathana et al., 2019

Where, (–): Information not available, (R), resistant; (S), susceptible.

Differential mungbean−YMV interaction appears the most probable reason for the identification of various types of resistance reactions to the YMD. The dominant MYMV resistance gene action indicates gain-of-function, while recessive inheritance signifies loss of host genes function which appears essential for virus infection, replication, and cell-to-cell movement (Diaz-Pendon et al., 2007). Weather parameters regulating whitefly activity is another very crucial factor for the viral disease expression under open field conditions (Sudha et al., 2013b). Since YMD resistance in mungbean was mostly reported controlled by digenic dominant interaction with some modifier genes, therefore the use of recombination breeding and delayed selection method should be more effective for the incorporation of YMD resistance (Mahalingam et al., 2018; Dikshit et al., 2020). The recessive nature of YMD resistance also emphasizes the significance of marker-assisted selection (MAS) for quick and precise YMD resistance breeding programs in mungbean (Chen et al., 2013).

## Insight About Whiteflies as a Vector and YMD Development

Whitefly (*Bemisia tabaci* Gennadius) (Hemiptera, Aleyrodidae), a polyphagous pest of Indian origin, causes severe damage to over 1,000 plant species, not only by sucking the plant sap but also as a vector of several viral diseases (Fishpool and Burban, 1994). It can transmit nearly 300 virus species of multiple virus genera including *Begomovirus* (~90%), *Carlavirus*, *Crinivirus, Closterovirus*, and *Ipomovirus* (4%) (Simon et al., 2003; Castillo et al., 2011; www.whiteflygenomics.org). The mouthparts of the whiteflies are designed to retain the virus through their stylet, while feeding on the phloem sap from the plant. After entering the vector, the virus moves in a persistent circulative manner (Czosnek et al., 2002) and during its next feeding on a healthy plant the virus is injected with salivary secretion. The virus circulates (do not replicate in the whitefly) from the foregut, midgut, hindgut, hemolymph, and finally to the salivary glands of the whitefly before their release into the plants (Fiallo-Olivé et al., 2020).

For acquisition and inoculation of virus through phloem sap, the vector requires at least 15 to 60 min and 15 to 30 min, respectively. However, 8 h of minimum latent period is a must between acquisition and inoculation, for successful transmission of viruses (Ghanim et al., 2001; Czosnek, 2008). Whitefly transmission ability is directly proportional to its acquisition access period (AAP) while gender and age of the vector also influences the virus transmission efficiency (Czosnek et al., 2002). The persistent mode depends on the minimum AAP and maximum duration of retention (generally 3 days for male and 10 days for female whiteflies) of the virions in the whiteflies.

Although, whitefly nymphs can get the virus from infected leaves, however, the virus cannot traverse to the eggs. Moreover, infectivity cannot be retained for the lifetime by either male or female whitefly (Karthikeyan et al., 2014). The interaction between the highly conserved virus CP and the receptors in the gut and salivary glands of the whitefly imparts *Begomovirus*-whitefly specificity, and any alteration in the virus CP also alters their vector preferences. Various proteins encoded by the whitefly like molecular chaperone proteins, HSP70 to assist the efficient circulative transmission of viruses (Brown and Czosnek, 2002; Varun and Saxena, 2017).

Murugan and Nadarajan (2012) reported no correlation between the presence of leaf trichomes in blackgram and whitefly activities and thus resistance to YMV, however, no such report is available for mungbean. Begomoviruses can negatively influence the longevity and fecundity of whiteflies to enhance their transmission; while whitefly behavior and feeding habits also influences the genetic composition and evolution of viruses (Varun and Saxena, 2017).

The globally accepted identification method of *B. tabaci* complex is the identification of divergence threshold of mitochondrial cytochrome oxidase subunit I (*mtCOI*) gene which was earlier considered at 3.5% (Dinsdale et al., 2010) and later changed to 4.0% (Lee et al., 2013). Sequence analysis of *mtCOI* has partitioned them into more than 41 morphologically indistinguishable groups or cryptic species (Dinsdale et al., 2010; De Barro et al., 2011; Mugerwa et al., 2018; Hu et al., 2018; Kanakala and Ghanim, 2019). However, these cryptic species do possess considerable variations for traits like host-range, insecticide resistance, and dispersing capability (Simon et al., 2003; Nair et al., 2017). In general, maximum whitefly diversity is reported from Asia. Of 11 genetic groups reported from India, Asia-I and Asia II-1 are found predominant with a significantly higher transmission efficiency of Asia-I (Anokhe et al., 2018).

The B-biotypes or Middle East-Asia Minor 1 (MEAM) 1 are found in arid, irrigated cropping system while Q-biotypes or Mediterranean MED species can adapt to greenhouse environments (Dinsdale et al., 2010; De Barro et al., 2011; Horowitz and Ishaaya, 2014). Recent whitefly whole genome sequencing has revealed that the Asia II-1 and Middle East Asia Minor 1 (MEAM1) species differ for the genes involved in virus transmission and insecticide resistance (Hussain et al., 2019). This indicates the need to generate more sequence information for different whiteflies biotypes across the world for holistic management of disease. Detailed studies on the whitefly-*Begomovirus* co-evolution in terms of their transmission, YMV-whitefly interactions and proteins associated in virus movement inside the whitefly can assist in the formulation of novel and more effective ways of YMV management (Varun and Saxena, 2017).

## Screening Methods and Varying YMD Resistance Expression in *Vigna*

Since mechanical transmission of YMV is not possible, therefore screening of mungbean for YMD resistance is mostly performed at the YMV hot spots. However, screening using viruliferous whiteflies and agroinoculation techniques which are more precise are on the rise. The details are discussed in this section.

### Screening of Genotypes at YMV Hot-Spots

The evaluation of mungbean against YMD under hot-spot conditions are carried out using infector-row technique in certain standard statistical experimental design. Generally, one row of a most susceptible spreader genotype of that region is sown after every two (Habib et al., 2007), three (Nair et al., 2017) or 10 rows of the test-genotypes (Sai et al., 2017) and also two rows of spreader may be planted all around the experimental area for having sufficient YMV load (Habib et al., 2007). Recommended cultural practices with no insecticide spray should be followed so as to encourage the whitefly population for sufficient infection and spread of YMD. Since whitefly starts infecting the plants soon after germination and YMD symptom is first visible during 2nd week after planting (continues till 6th week), therefore crop should be constantly watched for the presence of whitefly and YMD development. The disease can be scored as per the scale of Khan et al. (2007) when 90% of the infector rows express the YMD incidence and the genotypes can be categorized in various groups from resistant till susceptible (Selvi et al., 2006; Habib et al., 2007).

The major limitation under hot-spot screening is that the causative viruses and also the whitefly biotypes remain unknown (Shivaprasad et al., 2006). In addition, there is always chance of non-uniform disease development due to the varying whitefly population which simultaneously depends on the planting locations and season (Laosatit et al., 2020). Under field conditions, more whitefly built-up were reported at a higher temperature; whereas, high-rainfall and high-humidity results in a negative impact on the whitefly population (Rahman et al., 2006; Islam et al., 2008). Besides, a negative correlation between high-altitude regions with low-humidity and YMD incidence highlights the significance of various environmental factors influencing the YMD severity (Alam et al., 2014c).

### Screening Using Viruliferous Whiteflies

The screening of genotypes for YMD resistance under the net-houses using viruliferous whiteflies appears a better option (Shivaprasad et al., 2006). The whiteflies were first made viruliferous by force-feeding on YMV agroinfected mungbean plants for nearly 24 h, also known as acquisition access period (AAP) and these were then used for the inoculation of healthy plants for approximately 24–48 h, also known as inoculation access period (IAP). Whitefly is an extremely efficient vector as even a single viruliferous adult can transmit the YMV within 24 h of AAP and IAP (Malathi and John, 2009). Govindan et al. (2014) reported 10 viruliferous adult whiteflies after 24 h each of AAP and IAP causing YMD with 70.50% virus transmission efficiency, while 20 viruliferous whitefly adults after 48 h of AAP and 24 h of IAP has resulted in 85% virus transmission efficiency. Since YMD can be very effectively spread by very low densities of adult whiteflies; therefore, no correlation could be established between the number of whiteflies and YMD severity (Akhtar et al., 2011).

### Screening Through Agroinfection

As the YMV can only be transmitted by the whitefly vector, thus agroinoculation based genotypic screening is considered better option for the identification of YMD resistance sources (Sudha et al., 2013c). Agroinoculation in mungbean are performed on surface sterilized 2 d sprouted seeds (Jacob et al., 2003) grown in Hoagland’s solution by removing the seed coat and then pricking near the hypocotyl region and then immersing the pricked seeds in *A. tumefaciens* culture (Sudha et al., 2013b; Sudha et al., 2013c; Sai et al., 2017). After overnight incubation, seeds are washed using distilled water and sown in pots. Afterwards, the inoculated plants should be grown in a growth chamber with 16/18 h photoperiod, 25°C temperature and 60–70% of relative humidity (Karthikeyan et al., 2011). The appearance of YMD symptoms in the leaves can be noted from 7th to 12th day of inoculation, while infectivity (%) can be calculated based on the number of infected plants to the total number of inoculated plants (Sudha et al., 2015). Plants should be transferred to the greenhouse after 15 days after symptom appearance (Balaji et al., 2004). The biggest advantage with agroinoculation is that it creates uniform disease conditions than those of natural conditions and thus the symptoms are also easier to compare (Sudha et al., 2015).

A field-based screening of 78 mungbean genotypes for MYMV has identified 28 genotypes as resistant while on agroinoculation of same genotypes only 03 (ML1108, KMG189 and SP84) and 01 (ML818) genotypes were found resistant to the VA221 (KA30 DNA-A and KA22 DNA-B) and VA239 (KA30 DNA-A and KA27 DNA-B) strains, respectively (Sudha et al., 2013c). Thus, it was assumed that the resistance expressed at the field could be because of certain mechanisms preventing the viral entry into the plant through insect vectors.

**Figure 4:**
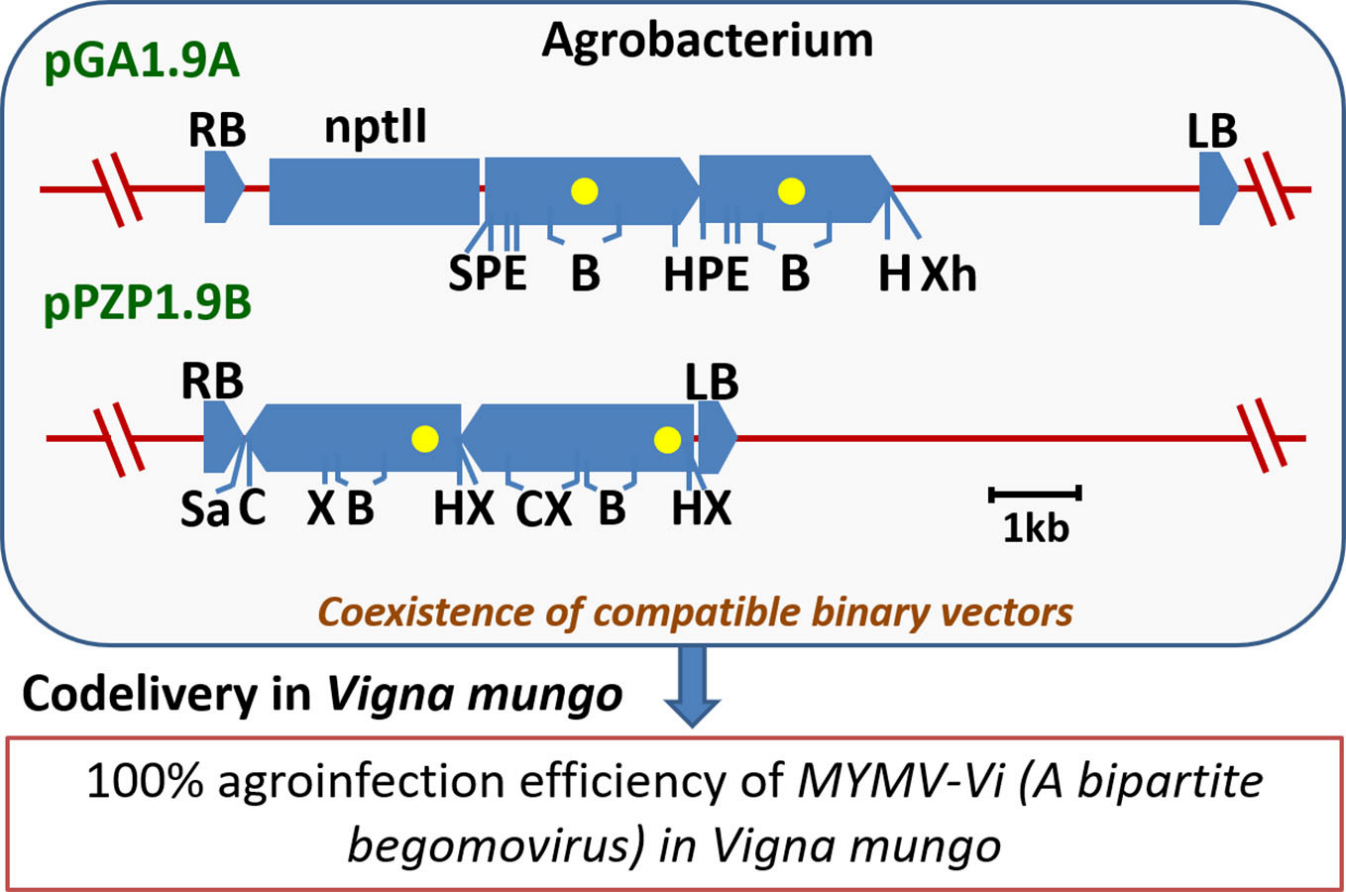
An outline of a simple and efficient, ‘single-strain agroinfection method’ of a bipartite begomovirus MYMV-Vi in *Vigna*. The linear maps of binary vectors represent MYMV-Vi partial tandem repeat regions of DNA-A (pGA1.9A) and DNA-B (pPZP1.9B) having full-length 1-mer portion and the 0.3-mer or 0.9-mer repeat portions of the virus as boxed arrows; Yellow dots: common region; RB and LB: right and left T-DNA borders, respectively; *nptII: neomycin phospho-transferase II*; *B, BamHI*; *C, ClaI*; *E, EcoRI*; *H, HindIII*; *P, PstI*; *S, SacI*; *Sa, SalI*; *X, XbaI*; and *Xh, XhoI* (Derived from Jacob et al., 2003).

Generally, for bipartite geminiviruses, the agroinfection is performed by mixing two *Agrobacterium* strains harboring partial tandem repeats of DNA-A and DNA-B components, independently. However, Jacob et al. (2003) reported a ‘single strain agroinfection method’ of a bipartite begomovirus, which employs a combination of binary vectors, pGA1.9A and pPZP1.9B having MYMV-Vi DNA-A and DNA-B partial tandem repeats, respectively in the same *Agrobacterium* strain. This method consistently gave 100% agroinfection in blackgram (**Figure 4**). Moreover, when viral load is minimal and also for the asymptomatic plants; real-time PCR assay should be opted over conventional PCR assays (Sudha et al., 2013c). Thus, not only understanding the YMV resistance mechanism, but also quantification of viral load in the virus challenged plants appears essential while evaluating the YMV resistance.

## Seed Borne Nature of YMVs

Begomoviruses are mostly confined to the phloem parenchyma and cambium, and rarely to mesophyll parenchymatous tissue, thus they can reach seed parts only till seed coat hilum (Rojas et al., 2005; Kothandaraman et al., 2016). However, the early symptom appearance as yellowing of the very first trifoliate leaf of the blackgram seedling in the field indicated the possibility of seed-borne nature of YMV. PCR amplicons sequencing, DAS-ELISA, immunosorbent electron microscopy, and confocal microscopy confirmed the presence of MYMV in the seed coat, cotyledon, and embryonic axes. However, the seeding growth tests revealed no YMD symptoms, though both DNA-A and B components of MYMV could be detected in 32% of the seedlings (Kothandaraman et al., 2016). It was speculated that the vigorous metabolic environment of seedling could be inhibiting the efficient build-up and translocation of the virus leading to no symptom. However, whitefly transmission of the virus was not demonstrated from the PCR confirmed symptomless seedlings.

On contrary, when seeds (with yellow patches on the seed coat) from MYMIV infected mungbean plants when used for PCR amplification do showed the presence of virus, but it could not be detected in the seedlings of PCR positive seeds, and the seed-borne nature of YMD in mungbean was ruled out (Naimuddin et al., 2016). Except for the report of Kothandaraman et al. (2016), there was no other report confirming or validating the seed-borne nature of YMVs in any other *Vigna* species. Thus, detailed analysis is still needed to confirm the exact mechanism of seed-borne nature of YMVs in different *Vigna* species.

## Biochemical Changes During Mungbean–YMV Interactions

**Figure 5:**
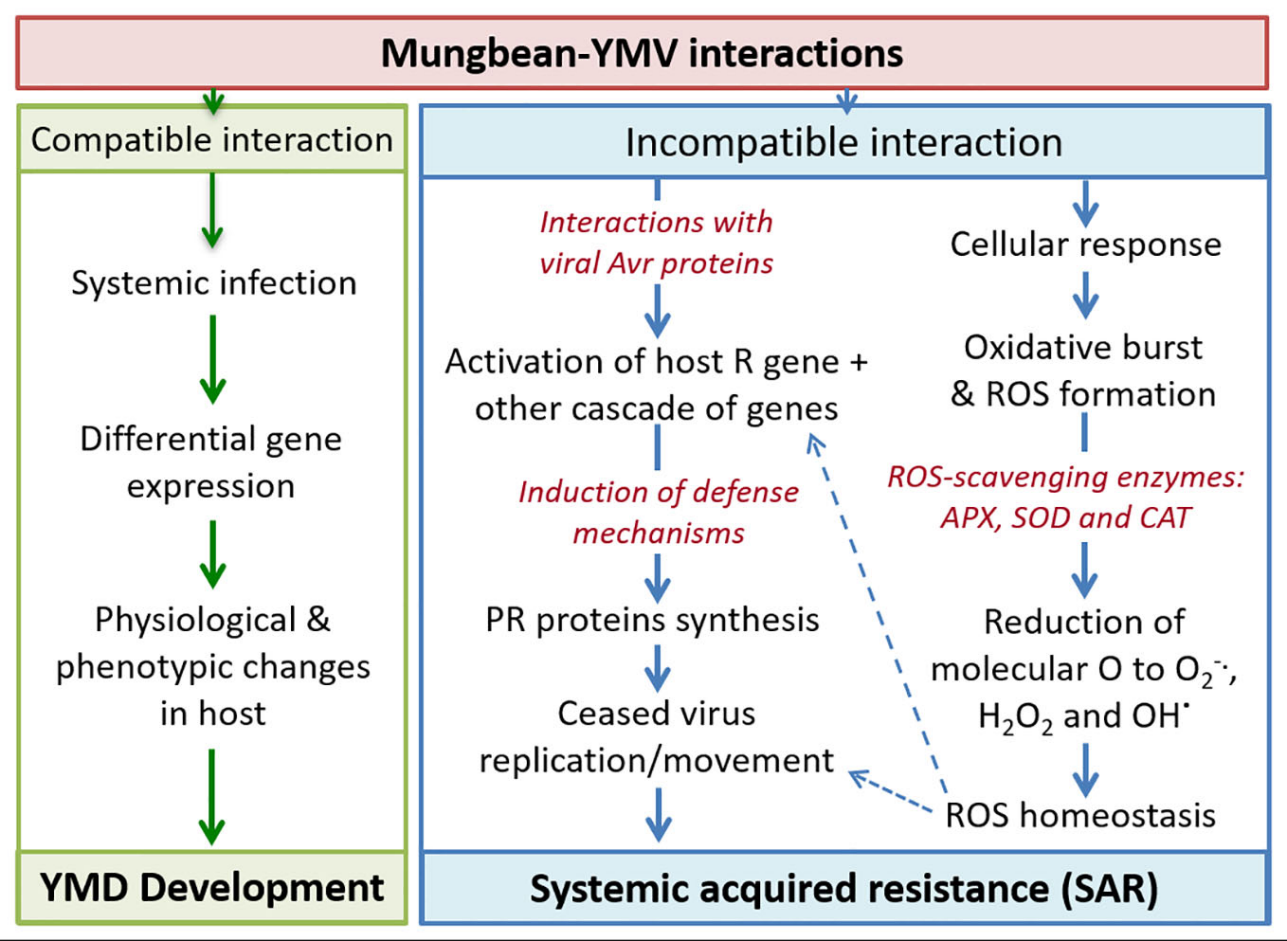
Schematic presentation of plant-virus interaction leading to disease development and resistance expression in mungbean. Where, Avr, Avirulence; R, Resistance; PR, Pathogenesis-Related; ROS, Reactive Oxygen Species; APX, Ascorbate peroxidases; SOD, Superoxide dismutases; CAT, Catalase.

Upon YMV infection, the compatible reaction results in systemic infection leading to symptom expression (Yang et al., 2007). During YMV-host incompatible reaction, the resistance gene expression gets activated upon interaction with viral avirulence (*avr*) proteins which then triggers a cascade of defence genes including pathogenesis-related (PR) proteins which are also associated with systemic acquired resistance (SAR) (Sels et al., 2008). All these ultimately results in the ceased replication and arrested movement of the YMVs (**Figure 5**). Various ROS-scavenging enzymes *viz*. ascorbate peroxidases, superoxide dismutases, and catalases are reportedly maintaining the ROS homeostasis in the plant cells which eventually inactivates the virus (Torres, 2010; Oliveira et al., 2012).

The relative expressions of defense and signal transduction associated proteins are important for the induction of YMD resistance. Photosynthesis pathway proteins, especially PS-II electron transport pathway are mainly affected in susceptible genotypes under YMV-stress. In addition, significantly elevated levels are recorded for phenolics, H_2_O_2_, and carbohydrates in YMV incompatible interaction over compatible reaction. The pathways associated with the induction of defense response carries various core proteins, of which ascorbate peroxidase, rubisco activase, and serine/glycine hydroxyl-methyltransferase are the nodal hub which results in defense response. Also, YMV resistance in blackgram was reported channelizing the carbohydrate flux towards the pentose phosphate pathway (Kundu et al., 2013). Thus, the role of various biochemicals (involved in the ROS homeostasis) in imparting YMD resistance in mungbean should be established in a gene-network derived pathway mode using high throughput transcriptomic studies, during both compatible and incompatible reactions.

## YMD Management Challenges and Opportunities

The YMD management using insecticides (to control whiteflies) has been considered effective, but due to the development of insecticide resistance in vectors, the disease is on the rise. In addition, excessive use of chemicals resulted in detrimental impacts on both environment and human health (Mishra et al., 2017). Several YMD management strategies for the sustainable management of YMD are thoroughly discussed in this section.

### The Management of Primary Hosts of YMV and Its Vector

Eradication of primary hosts of YMV such as perennial weeds and summer whiteflies will facilitate YMD management (Malathi and John, 2009; Karthikeyan et al., 2014). The significant YMV hosts include, *V. radiata*, *V. mungo*, *V. aconitifolia*, *V. unguiculata*, *Cajanus cajan*, *Glycine max* and *Phaseolus vulgaris* (Varma et al., 1992; Karthikeyan et al., 2004; Qazi et al., 2007; Karthikeyan et al., 2014). Alternatively, other leguminous hosts namely, *V. hainiana* and *V. trilobata*, have also been confirmed as natural hosts (Naimuddin et al., 2011a; Ramesh et al., 2017b). Besides, ‘infected tolerant plants’ or ‘symptomless carriers’ may also act as a virus-host.

Managing whiteflies is quite complex, as they attack in hundreds and even one attack is enough for the severe weakening of a plant. In the Northern and Southern Indian conditions, two indigenous cryptic species *viz*. Asia II-1 and Asia II-8, respectively are reported predominant (Nair et al., 2017). Since whitefly species differ significantly in its sensitivity to various insecticides, therefore inclusive information about the abundance of whitefly species of any region is essential for the rational use of insecticides (Horowitz and Ishaaya, 2014). The application of systemic insecticide combinations at the early growth stage proved effective for whitefly management, as it kills the vector and simultaneously protects the plant against further attack (Wang et al., 2009; Dubey and Singh, 2013). Also, field-sanitation, plucking of infested leaves, water-sprays, avoiding an excess of nitrogen fertilizer are also recommended to curb the whitefly population (Karthikeyan et al., 2014). In addition, 8 h of seed hydro-priming was reported effective for lowering the incidence and severity of YMV infection in mungbean (Rashid et al., 2004).

### Resistance Sources for YMD in Mungbean and Blackgram

The YMD resistance is generally assessed by the appearance of symptoms using a commonly accepted disease scoring scale (Khattak et al., 2008; Iqbal et al., 2011; Panduranga et al., 2011). However, while selecting any genotype as resistant, utmost care should be taken and any symptomless carrier should never be used in the YMV resistance breeding program as a resistance source. Therefore, the resistance sources screening under open field conditions should also include the simultaneous identification of viral strains (Karthikeyan et al., 2014).

There are abundance of reports stating absolute YMD resistance among certain mungbean lines, but most of them were poor yielder (Pathak and Jhamaria, 2004; Salam et al., 2009). Generally, the mungbean germplasm having good yield potential is reported susceptible to the YMD (Khattak et al., 2008; Akhtar et al., 2011). Success has been achieved *via* shuttle breeding program between the World Vegetable Center (AVRDC) and NIAB (Nuclear Institute for Agriculture and Biology-Pakistan), which has resulted in the development of several mungbean varieties having YMV resistant (AVRDC, 1995; Khattak et al., 2008).

Most of the reports about the identification of YMD resistance sources in mungbean across the world are based on field screening (**Table 2**). A few Indian mungbean genotypes like IPM-02-03, PDM-139, Pusa0672, and HUM16 are reported resistant by different workers under different field conditions in different years (Asthana, 1998; Datta et al., 2012; Paul et al., 2013; Mohan et al., 2014; Subedi et al., 2016).

**Table 2:** A timeline of mungbean and blackgram genotypes, reported as YMD resistant and susceptible by different researchers.

S. No.	Resistant genotypes*	Susceptible genotypes	Reference
	**Mungbean**		
1.	Moong No. 54, P364-68, P366-68, 15229, L24-2, LM-168, LM-170, -214, -356, -392, -404, -171, 15225, 15227, MIr3, ML-1, -5, -6, -9, Tarai local, L-80, LM294-1	–	Virmani et al., 1976; Singh et al., 1977; Pandya et al., 1977; Singh, 1980b
2.	PDM-11, PDM-54, PDM-84-143, NM92, ML-5, Var.6601, EC30072, K141, LGG424B, LM108B	VC2272, Pusa Baisakhi, VC1560D, VC3902A, Berken, Emerald	Asthana, 1998; Khattak et al., 2000; Manivannan et al., 2001
3.	RU2229, VBG86, 2KU54, VBG89, SU16, ML-5, MUM-2	–	Pathak and Jhamaria, 2004
4.	GG-89, GG-39, TM-98-50, TM-97-55, Co-5	Chinamung	Salam et al., 2009
5.	PDM-139 (Samrat), IPM-02-03, Pusa0672, HUM16, MB-57, MB-58, Pant Mung-2	–	Datta et al., 2012; Paul et al., 2013; Kumar and Kumar, 2014; Subedi et al., 2016
6.	EC 398897, TM-11-07, TM-11-34, PDM-139, IPM-02-03, IPM-02-14, Pusa0672, Pusa0871, CO-7, MH-521	RMG341, LM702A, PLS265	Mohan et al., 2014
7.	NM-2011, -2006	M-6, 8010, 8011	**Binyamin et al., 2015
8.	BRM-325, -345, -363, -364, -366, NM-2011	BRM-349, -350	**Ahmad et al., 2017
9.	KMP-13, 19, 20, 22, 23, 24, 40, 45, MLGG-8, WGG-42	–	Bhaskar, 2017
10.	CM15-7-13, -2-9, -3-10, -3-8, -8-10, -5-6	CM15-5-23, -2-1, -2-3, -3-18, -1-1, CM14-2-12	Sai et al., 2017
11.	IPM02-03, KM2241, PDM139, Pusa0672, HUM16, ML1464, TARM-1	HUM-12, LG-460, K-851, Pusa Vishal, COGG 902, MH84-1, SML-1455, China mung, Kopergaon	Bhanu et al., 2017b
12.	NM94, ML 1628	KPS2	Nair et al., 2017
13.	SML1815, MH421	VBN(Gg)3, VBN(Gg)2, LGG-460, RMG10-28, TM96-2	Mahalingam et al., 2018
14.	Sonali × *V. radiata* var. *Sublobata* derivatives (BS13 to 20, -24, -26, -27, -35, -47, -49, -55, -57); SM12-78, Sonali, SPM-13-5, SM12-80, SM13-10, SM13-46, SPM13-34, KM11-557 (KM-11-PM4), TGM-3, SM12-56	Sonali × *V. radiata* var. *Sublobata* derivatives (BS2, BS6); Pusa Vishal, SM13-14	Singh et al., 2018
15.	AVMU-1698, -1699, -16100, -16101, KPS2	Harsha, NM-94	Nagaraj et al., 2019
**Vector resistance/tolerance**			
16.	G65, IM170, LM47, 141, 170, 364, M170, ML1, 3, 5, 6, 7, 15, 24, 186, 192, 194, 195, 197, 235, 337, 423, 428, 711P 131, 242, 290, 292, 293, 325, 364, T44, 192-1, 10866, 11148, 15127	–	Shanmugasundaram,1996
17.	NM92	–	Khattak et al., 2004
18.	ML803, ML839, PDM91-249, PBM5	–	Yadav and Dahiya, 2000
19.	TMB36, RMG1004	–	Singh and Singh, 2014
20.	ML-1265, -1229, -1265, -1774, -1779		Cheema et al., 2017
	**Blackgram**		
21.	KARS114, IPU245, PGRU9518, KUG-50	T9, IVU-486, PantU-02-11, UG-4, VKG-30-28, IPU-99-23, LBG-752	Singh et al., 2008
22.	PU-35, -U31	ADT-5, LBG-623, IU-98843, -652, -835, -834, -861, -943, LBG-752, -685, -402, -645, -17, -22	Gopi et al., 2016
23.	PU-1075, -31, -205	LBG-623, -645, -685, IC110790, IC145202, IC1575, IC164118, IC20880, IC59718, IC61106, IC61603, IC73306	Vishalakshi et al., 2017
24.	RSU03, TU22, Pant-U-31, RSU06	–	Babu et al., 2019
25.	VBG11-053, LBG808, CO6, VBN4, VBG10-019, R15-006, R15-011, R15-009, VBG11-010, KU52, KU24, ACM14001, ACM015-30, ACM015-29	R-15-008, -15-001, LBG645, KU-003, -42, -50, -51, ACM015-14	Tamilzharasi et al., 2019
26.	KU96-3, NDU12-1, NIRB-002, -003, -004	AAU34, AKU-10-4, -11-15, -15, -7-4, -7-1, CO5, COBG-10-06, -11-02, -11-03, LBG-623, -645, MU46, NUL-2-5, -138, PDU1, TAU-1, -4, TU17-4, -26, VBN(BG)4	Kumari et al., 2020

Where, DAS, Days after sowing; *Field Screening; **Reported from Pakistan while all others from India.

Notably, the genotype found resistant in one location may not be resistant under other locations, as the resistance is viral strain specific. Thus, while selecting the resistant parent for YMD resistance breeding, it is advised to first screen all the genotypes at any given location, and depending on the results, crossing programme should be designed. Confirmation of YMD resistance using agro-inoculation of age no type appears as the best option, as it results in significantly uniform disease expression (Sudha et al., 2013c).

### Wild Relatives and Wide-Hybridization for YMD Resistance

Some wild relatives of mungbean, like a few accessions of *V. radiata* var. *sublobata* (Singh and Ahuja, 1977), *V. trilobata*, *V. mungo*, and *V. umbellata* (Pandiyan et al., 2008), have been reported as YMD resistant. Seven *Vigna* accessions *viz*., *Vigna* synthetic allotetraploid, *V. umbellata*, *V. mungo* var. *mungo*, *V. trilobata*, *V. trinervia* var. *bourneae*, *V. radiata* var. *sublobata* and *V. dalzelliana* were reported free from YMD (Gautam et al., 2014). Also, certain accessions of *V. umbellata* were found resistant to a few isolates of MYMV, which can be used for the transfer of MYMV resistance into *V. radiata* and *V. mungo via* inter-specific cross (Sudha et al., 2015).

On contrary, MYMIV was recorded as the predominant virus causing YMD in 40 accessions of different wild species of *Vigna*. Likewise*, V. hainiana* (IC331450) was found infected with MYMV (Gautam et al., 2014). Naimuddin et al. (2011a) also reported natural infections of MYMIV in two wild *Vigna* species, *viz*. *V. hainiana* (IC*-*331615) and *V. trilobata* (IC-331436) under Indian conditions. Thus, care must be taken while selecting the wild *Vigna* species for the transfer of YMD resistance in mungbean through wide-hybridization.

At present, the World Vegetable Center (AVRDC) holds nearly 12,153 *Vigna* accessions which is the largest collection, representing a vital resource for inter-specific hybridization (Kim et al., 2015). The cross-compatibility among *Vigna* species is not very well defined and for widening the genetic base of *V. radiata*, the crossing using secondary gene pool including blackgram (*Vigna mungo* (L.) Hepper), rice bean [*V. umbellata* (Thunb.)], *V. radiata* var. *sublobata* and *V. trilobata* have been attempted with some success (**Table 3**). Unfortunately, wide hybridization in mungbean recorded severe cross-barriers like development of a few, small and mostly non-viable hybrid seeds, embryo death or hybrid sterility, incompatibility in chromosomal pairing and chromosome elimination (Pandiyan et al., 2012; Sudha et al., 2013a; Pratap et al., 2018). DNA marker analysis has also shown severe segregation distortion and chromosome elimination in an F_2_ population derived from a cross between mungbean and rice bean (Sudha et al., 2013a).

**Table 3:** Wide-hybridization for the creation of YMD resistance.

S. No.	Wide hybrid/Interspecific hybrids	Remarks	Reference
1.	*V. radiata* var. *sublobata* Roxb. Verde. × *V. radiata*	YMD resistance	Singh and Ahuja, 1977
2.	*V. radiata* × *V. radiata* var. *sublobata*	-Do-	Singh and Sharma, 1983
3.	*V. sublobata* and *V. mungo* to *V. radiata*	-Do-	Pal et al., 2000
4.	*V. radiata* var. VRM (Gg) × *V. umbellata* (Rice-bean)	YMD resistance	Pandiyan et al., 2008
5.	*V. radiata* (NM92) × Bruchid-resistant *V. radiata*ssp. *sublobata* (TC1966)	Mapped 03 major QTLs for resistance to MYMIV on LG 9	Chen et al., 2013
6.	*V. radiata* (L.) Wilczek × *V. umbellata* (Thunb.)	YMD resistance and SCAR marker development from ricebean	Sudha et al., 2013a
7.	*V. synthetic* allotetraploid	YMD resistance	Gautam et al., 2014
8.	Blackgram (PS1) × ricebean (RBL-1, -6, -35, -50)	-Do-	Sehrawat et al., 2016
9.	Ricebean genotype, RBL1 x mungbean genotypes, TM 96-2 and K 851	-Do-	Bhanu et al., 2017a
10.	*V. radiata* (SML668 and SML832) × *V. mungo* (Mash114 and Mash218)	-Do-	Lekhi et al., 2018
11.	*V. radiata* (VBN(Gg)2) × *V. stipulacea*	-Do-	Chitra et al., 2018

Measures such as the use of mentor pollination, embryo rescue, and hormonal manipulations are reported to increase the success of interspecific crosses. To overcome the cross-compatibility problem of mungbean with rice bean, use of either 100 ppm E-Amino Caproic Acid (EACA), or *V. radiata* var. *sublobata* as a bridge species was reported successful (Kumar and Kumar, 2014). The hybrids between the cross of *V. mungo × V. radiata* were obtained through sequential embryo rescue (Gosal and Bajaj, 1983; Verma and Singh, 1986). In India, till now only three mungbean varieties namely, HUM1, Pant Moong4 and IPM99-125 having a high level of YMD resistance could be released using mungbean × blackgram crosses. Thus, more concerted efforts are required to not only overcome the cross-compatibility barrier but also to prevent chromosome elimination while attempting for the wide hybrids.

### Mutation Breeding

Mutation breeding is an instant way of accelerating the genetic variation for various traits including YMD resistance in crop plants. In mungbean, 10–30 KR dose was found quite effective for getting the desirable mutants for traits like earliness, synchronous maturity, and YMD resistance (Singh and Chaturvedi, 1981). While performing the mutation breeding, the breeders generally select one or a few target traits for the improvement purpose. Single plant selections were performed under disease pressure conditions during M2 and onwards generation to find the plant(s) with YMD resistance and high yield through the selection of various other traits like fertile branches per plant, pods per plant and seed yield per plant, etc. These mutant lines may be released as such as a variety or the traits may be incorporated in other varieties through backcross breeding (Pratap et al., 2020).

At Nuclear Institute for Agriculture and Biology (NIAB, Pakistan) the mungbean improvement was initiated in 1970s with major focus to create variations through induced mutations (gamma irradiation) and hybridization, to develop high yielding and YMV resistant varieties (Haq, 2009). NAIB in collaboration with World Vegetable Center started the crossing program using a mutant YMV resistant line with KPS1, which resulted in the development of to two advanced YMD-resistant lines namely NM-92 (NIAB Mungbean-1992) and NM94 (Ali et al., 1997) which were introduced to various countries in South Asia (Shanmugasundaram et al., 2009). Two very popular summer mungbean cultivar of India, Pusa Vishal and SML-668 was also derived from NM-92 and NM-94 respectively, through selection for YMD resistance and synchronous maturity. NM-92 also became very popular in other countries like Bangladesh and Myanmar (Brar et al., 2006).

Several mutant varieties in mungbean have been developed across the world which are both high yielding and also resistant to many biotic stresses including YMD (**Table 4**). Based on field scoring, Vairam et al. (2016) have identified 05 mutants (*viz*., M5, M18, M26, M70, and M71) which in M3 generation showed YMD resistance. Thus, the mutation breeding approach looks promising not only for the creation of YMD resistance but also for the yield improvement without severely altering the existing genetic architecture (Vairam et al., 2016).

**Table 4:** List of YMD resistant mungbean varieties/advanced breeding lines developed through the mutagenesis approach.

S. No.	Variety/Advanced breeding material	Mutagenic treatment	Parent variety/Cross	Reference
1.	BINA Moog-2 (MC-246)	Gama-rays	MB-55 (Mutant MB-55 (4) × V-2273)	Ahmed et al., 1995
2.	Pant Moong-2	10 KR gama-rays	ML-26	Kharakwal, 1996
3.	BM-4	EMS (0.15%)	T-44	
4.	MUM-2	EMS (0–2%)	K851	Gupta, 1996
5.	TMB-37,	Gamma rays	Kopergaon × TARM-2	D’Souza et al., 2009
6.	TJM-3	-do-	–	
7.	NIAB Mung 2006	EMS	–	Haq, 2009
8.	Mutant SML-668	Gama-rays (600 Gy)	SML-668	Reddy, 2009
9.	Chai Nat 72 (CN 72)	Gamma-rays(600Gy)	Khampang Saen 2 (KPS2)	Ngampongsai et al., 2009
10.	Pant Moong	100 Gy	–	Auti, 2012
11.	ML-26-10-3	Gama-rays	–	
12.	sTARM-1, TARM-2	30 kR gama-rays	S-8	
13.	TARM-18	-do-	S-8 (PDM54 × TARM-2)	

Where, EMS, ethyl methane sulphonate; kR, kilorad.

### Marker Assisted Selection (MAS)

The identification of tightly linked molecular marker(s) with the YMV resistance gene and screening of genotypes through MAS can augment the selection precision for the YMD resistance (Laosatit et al., 2020). Additionally, the recessive expression of YMV resistance also highlights the importance of MAS for mungbean breeding programs (Chen et al., 2013). Although, a large number of DNA markers reported linked with YMD resistance in both mungbean and blackgram (**Table 5**), but not yet very successfully used in the breeding programme, possibly due to the poor linkage or parental polymorphism (Selvi et al., 2006; Souframanien and Gopalakrishna, 2006).

**Table 5:** List of molecular markers linked with MYMD resistance in mungbean and blackgram.

S. No.	Marker	Marker details	Genotypes	Remarks	Reference
	**Mungbean**				
1.	RAPD	OPAJ20 (ACACGTGGTC)	NM92	MYMV; RILs	Lambrides et al., 1999
2.	RAPD	OPS7 (TCCGATGCTG)	ML267(R) × CO4 (S)	900 bp; F_2_; MYMIV	Selvi et al., 2006
3.	24 RGA cowpea primers	–	06 each of MYMV R and S genotypes	R and S genotypes into nearly distinct cluster	Narasimhan et al., 2010
4.	SCAR	MYMVR-583 (Fwd: GTGATGCACACGGTTACGGT; Rev: GGTGACGCAGTCCATACAAATT);	RIL: TM-99-37 (R) × Mulmarada (S)	2,023 bp, 6.8 cM, MYMV	Dhole and Reddy, 2013
5.	RAPD	OPBB 05 (GGGCCGAACA)	VBN(Gg) (S) × KMG189 (R)	260 bp; MYMV	Karthikeyan et al., 2012
6.	SSR	–	RILs: NM10-12-1 (R) × KPS2 (S)	05 QTLs (qMYMIV1-5), 6.24–21.93% PVE, MYMIV	Kitsanachandee et al., 2013
7.	SCAR, AFLP and SSR	*MYMIVr 9_6.4* (AFLP: m4pcc585); *MYMIVr 9_25* (SSR: DMB158, Fwd: TGGAAAATTTGCAGCAGTTG; Rev: ATTGATGGAGGGCGGAAGTA)	NM92 (R) × TC1966 (Wild and S)	04 QTLs, 1. *MYMIVr7_104* (LG:07; SCAR); *2. MYMIVr 8_48.8* (LG:08; AFLP); 3*. MYMIVr 9_6.4* (LG:09; AFLP); *4. MYMIVr 9_2 5* (LG:09; 59% PVE; SSR); MYMIV	Chen et al., 2013
8.	RAPD	UBC499 (GGCCGATGAT)	BL849 (R) × Chinamung (S)	700 bp; RILs	Holeyachi and Savithramma, 2013
9.	SSR	CEDG275 (Fwd: CACACTTCAAGGAACCTCAAG; Rev: GTAGGCAACCTCCATTGAAC), CEDG006 (Fwd : AATTGCTCTCGAACCAGCTC; Rev: GGTGTACAAGTGTGTGCAAG), CEDG041 (Fwd: GCTGCATCTCTATTCTCTGG; Rev: GCCAACTAGCCTAATCAG), VES0503 (Fwd : CGCTTTTGTAGGATTGGAACA; Rev: TGAAGGATGAGGGGAAGATG)	BARI mung 1 (S) × BARI mung 6 or NM94 (R)	F_2_ and BC_1_F_1_; 02 QTLs, (i) *qMYMIV2*.1 on LG02 (31.42-37.60% PVE) between CEDG275 and CEDG006 makers; and (ii) *qMYMIV7*.1 on LG07 (29.07-37.6% PVE) between CEDG041 and VES0503 markers	Alam et al., 2014b
10.	SCAR	(i). CM815 (Fwd: CGACTCACTATAGGGCGAATTG; Rev: AGCTTGGCGTAATCATGGT) (ii). CM9 (Fwd: TCCCGCTTTCCATGTGCAAG; Rev-ATGTTTGGGGAAAGCGGGAA)	KMG189 (R) × VBN(Gg)2 (S)	(i). CM815, 5.56 cM, 695 bp; (ii). CM9, Co-segregate, Chromosome 03, 306 bp	Sai et al., 2017
11.	RAPD	(i) OPBE9 (CCCGCTTTCC); (ii) UBC815 (CTCTCTCTCTCTCTCTG)	-do-	(i). OPBE9, 306 bp, cosegregate; (ii) UBC815,707bp, 5.56 cM	Sai et al., 2017
12.	SSR, STS	(i) VrD1 (Fwd: CAGCTTCTTGTTCTTGCTCC; Rev: CGAATGTGCACAGGTGGTGT), (ii) CEDG228 (Fwd: GTCGTTTCCGGAAACTGTTC; Rev: GATCCGAACCTCTTTCTG C), (iii) CEDG044 (Fwd: TCAGCAACCTTGCATTGCAG; Rev: TTTCCCGTCACTCTTCTAGG), (iv) STSbr1 (Fwd: CAGAAAACAAATCACAAGGC; Rev: GTAAGCATTGAAAAAGGG TG)	Sonali × *V. radiata* var. *sublobota*	Linked markers: VrD1, CEDG228, CEDG044 and STSbr1 with R^2^ = 6.0, 8.0, 11.33 and 18.0, respectively, MYMV	Singh et al., 2018
13.	RAPD	OPP 07_895_	–	Only BSA based identification	Dharajiya and Ravindrababu, 2019
14.	SNP	*–*	RILs (*V. radiata* × *V. umbellate*)	A QTL on chromosome 4, 10.11–20.04% PVE	Mathivathana et al., 2019
15.	SSR	CEDG293, DMB-SSR008 and DMB-SSR059	Association mapping panel of 127 genotypes	QTLs on LG 2, 4, 9; 11–14% PVE, MYMIV	Singh et al., 2020
	**Blackgram**				
16.	RGA-VMYR1	*RGA-1-F-CG*: AGTTTATAATTCGATTGCT; *RGA-1-R*: ACTACGATTCAAGACGTCCT	VM-1 to VM-7	VMYR1 (*RGA-1-F-CG*;*RGA-1-R*), 445 bp; 6.5 cM; MYMIV	Basak et al., 2005
17.	SCAR	*SCAR*_ISSR811_ (*YMV1-Fwd :* GAGAGAGAGAGAGAGACAAAG; *YMV1-Rev :* GAGAGAGAGAGAGAGACAGGA)	VM-1 to VM-7	*SCAR*_ISSR811_ (*YMV1*), 1,357 bp, 6.8 cM; RILs; MYMIV	Souframanien and Gopalakrishna, 2006
18.	SSR	CEDG_180_ (Fwd : GGTATGGAGCAAAACAATC; Rev : GTGCGTGAAGTTGTCTTA TC)	CEDG_180_	CEDG_180_, 12.9 cM, LG10, MYMIV	Gupta et al., 2013
	**Mungbean and Blackgram**				
19.	RGAs (YR4)	YR4 (*RGASF1*-GGNAAGACGACACTCGCNTTA; *RGASR1*-GACGTCCTNGTAACNTTGATCA)	–	YR4, 456 bp, both mungbean and blackgram; partially linked; MYMIV	Maiti et al., 2011
20.	RGAs (CYR1)	CYR1 (*RGA22F2:*GGGTGGNTTGGGTAAGACCAC; *RGA24R2:*NTCGCGGTGNGTGAAAAGNCT)	–	CYR1, 1,236 bp, both mungbean and blackgram, co-segregation, MYMIV	Maiti et al., 2011

Where, (–) means information not available.

#### Markers Linked With the YMD Resistance

Linkage map comparisons have revealed same linkage group (could be the same locus) for three major QTLs imparting YMD resistance viz., MYMIVE9_25, qMYMIV4/qMYMIV1, and qMYMIV2.1 from the genotypes NM92, NM10-12-1 and BARImung6, respectively (Kitsanachandee et al., 2013; Alam et al., 2014b). Interestingly, the resistance in these genotypes was derived from a common genotype 6601 (Laosatit et al., 2020). Thus, fine-mapping and cloning of the region should be attempted on priority for these QTLs for finding the functional details of this region.

**Figure 6:**
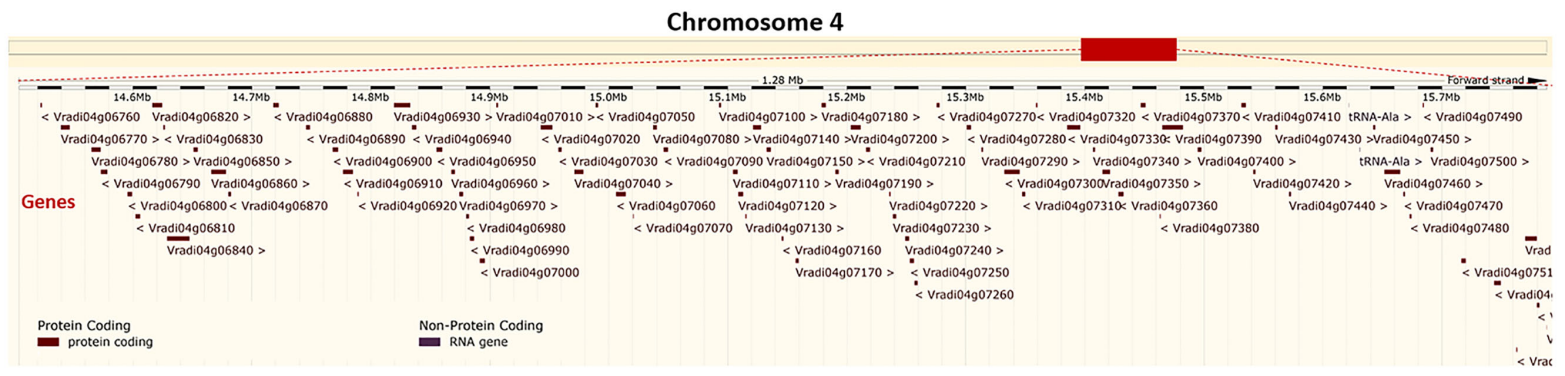
Physical location of a major QTL (qMYMV4_1) on the mungbean chromosome 4: 14,504,302-15,788,321. This region possesses 18 candidate genes imparting YMD resistance (Derived from: Mathivathana et al., 2019; https://plants.ensembl.org/Vigna_radiata/Info/Index).

Although ricebean is nearly immune to YMD (Sudha et al., 2015), yet due to low cross-compatibility this is occasionally used for the transfer of YMD resistance to mungbean (Sudha et al., 2013a; Bhanu et al., 2017b). Recently, QTL mapping of YMD resistance gene(s) using a RIL population (Mungbean-VRM (Gg)1 × Ricebean-TNAU RED) through GBS has revealed 05 QTLs having PVE from 10.11 to 20.04%. One major QTL qMYMV4_1 was found located in a 1.2Mb (14,504,302–15,788,321) region on mungbean chromosome 4 having 83 annotated genes of which 18 are considered as candidate genes (**Figure 6**) imparting resistance (Mathivathana et al., 2019). Since this is a big region, therefore adding more markers to this region will help in reducing the number of candidate genes for YMD resistance. However, the number of QTLs identified for the YMD resistance in TNAU RED is contrary to the fact that the resistance in ricebean is under the control of a single recessive gene (Sudha et al., 2013b).

However, Sai et al. (2017) have identified the MYMV resistance genes on chromosome 3 (using SCAR marker CM9); whereas, Yen et al. (2016) have reported several MYMV linked SNPs on the mungbean chromosomes 2, 5, 7, 9, and 10 (using CEL-I nuclease-based genotyping) (Yen et al., 2016). Till now, the chromosomal location of other markers linked with MYMV/MYMIV resistance genes or QTLs in mungbean are not yet worked out using integration studies. The details of markers linked with the YMD resistance genes/QTLs in mungbean and blackgram are presented in **Table 5**.

The detail gene mapping for the YMD resistance revealed that the genes imparting resistance to MYMIV (at least 02 loci) and MYMV in mungbean are different (Laosatit et al., 2020). Similarly, Alam et al. (2014b) also reported a SCAR marker (MYMVR-583) linked to a recessive gene imparting MYMV resistance in the genotype TM-99-37; but this marker was found not associated with the QTLs for the MYMIV resistance.

#### Candidate Gene for YMD Resistance

In both *V. mungo* and *V. radiata*, based on the role of ‘R genes’ in imparting plant virus resistance, the RGA markers (YR4 and CYR1) are reported completely linked with the resistance to MYMIV, suggesting that CYR1 could be a part of the candidate disease resistance gene (Pal et al., 2007; Maiti et al., 2011). Interestingly, CYR1 is also found associated, but not completely linked with MYMIV resistance in mungbean, indicating that the gene(s) for the resistance is not same and more than one locus is involved in imparting the resistance. Full-length sequence analysis of blackgram R gene CYR1 revealed it as 1,176 amino acids protein of non-TIR-NBS-LRR subfamily which by interacting with MYMIV-CP may act as a signaling molecule for recognizing the effector molecule of the pathosystem imparting resistance (Maiti et al., 2012).

Recently, BLASTN analysis of the CYR1 gene and linked SSR marker sequences for MYMIV resistance in NM10-12-1, NM92, and BARImung6 on the reference genomes of mungbean and azuki bean (*V. angularis*) showed that the CYR1 gene and other QTLs are present on different chromosomes (Laosatit et al., 2020). This has again reconfirmed that the resistance to MYMIV in mungbean and blackgram is different. The presence of different YMD resistant genes between mungbean, blackgram, and ricebean allows developing more-durable resistant genotypes *via* gene pyramiding.

#### Validation of Markers Linked With YMD Resistance

Of four markers (*viz*. VMYR1, YR4, CYR1, and SCAR_ISSR811_) reported linked with the YMD resistance when tested in a set of 14 blackgram genotypes revealed validation of three markers (YR4, CYR1, and SCAR_ISSR811_); while the marker VMYR1 produced monomorphic expression (Sowmini and Jayamani, 2014). Further, Binyamin et al. (2015) showed validation of two SCAR markers in 15 mungbean genotypes, which were reported linked with the MYMV resistance gene in both mungbean (Dhole and Reddy, 2013) and blackgram (Souframanien and Gopalakrishna, 2006). There are quite a good number of DNA markers known linked with the YMD resistance in mungbean and blackgram (**Table 5**), which still needs validation in a diverse set of genotypes. Such marker validation studies will not only help in speeding up the introgression of YMD resistance in different backgrounds, but also quick development of YMD-resistant genotypes without the need for artificial screening.

### Pathogen-Derived Resistance (PDR) Based Strategy

PDR refers to the ectopic expression of viral genomic sequences as RNA or protein, to impart resistance against the homologous (sequence wise related) or heterologous (unrelated) viruses, which can be deployed for expressing varied functional or dysfunctional YMV genes like coat protein (CP), protease, membrane protein (MP), replicase, etc. in mungbean, or gene silencing technology may also be used (Karthikeyan et al., 2014). In geminiviruses, CP, and Rep gene expression are mostly used for PDR (Kunik et al., 1994), but use of this technology in blackgram or mungbean is not yet successful due to their recalcitrant nature to *Agrobacterium*-mediated transformation.

Shivaprasad et al. (2006) in tobacco leaf disc assay showed MYMV genes-based PDR using CP, Rep-sense, Rep-antisense, T-Rep, NSP, and MP genes. Similarly, the effect of AC4-sense and AC4 hpRNA genes on MYMV DNA accumulation in tobacco leaf-disc assay has also revealed the potential of the AC4 hpRNA gene in imparting YMD resistance (Sunitha et al., 2013). However, the blackgram did not express any YMD resistance, when an MYMV derived DNA-A bidirectional promoter was used to activate PTGS against YMD (Pooggin et al., 2003). In another study, when mungbean plants were inoculated with infectious MYMIV clones containing the complementary-sense gene (ACI) encoding Rep, showed 64% infection (Haq et al., 2010). However, when co-inoculation was performed with the Anti-Rep construct, both symptom severity and infection percentage become negligible. The deletion in the CP amino-acids at N0 (75 and 150) of MYMIV has found affecting both systemic spread and pathogenicity (Haq et al., 2011), while agro-inoculation of the CP hairpin construct (Cphp) reported preventing the viral pathogenesis in mungbean (Kumari and Malathi, 2012). Kumar et al. (2017b) demonstrated RNAi-derived resistance to MYMIV in cowpea, where agro-infection of transgenic lines expressing *AC2-*hp and *AC2*+*AC4-hp* RNA showed nearly absolute resistance. These lines also reported accumulating transgene-specific siRNAs and very low level of viral DNA titers. In the era of rapid biotechnological advancements, very soon PDR will become a reality for YMD management in *Vigna*.

### Management of Single and Multiple Viral Infections

Since single and multiple viral infections are quite common under open field conditions. Mixed infections with MYMIV, GBNV, and ULCD were reported in blackgram which varied in different cultivars and seasons of the different year (Biswas et al., 2009). Thus, understanding the pattern of mixed viral infection in *Vigna* crops in different seasons will help in the identification of various factors leading to the multiple viral infections and ultimately help in the planning of better management strategies (Biswas et al., 2009).

### Scope of CRISPR-Cas9 Technology for the Imposition of YMD Resistance in *Vigna*

CRISPR (clustered regularly interspaced short palindromic repeat)–CRISPR associated 9 (CAS9) or CRISPR/Cas9 technology has been deployed to engineer the plants and confer resistance against begomovirus infection by using sgRNAs designed to target viral genomic DNAs (Khatodia et al., 2016; Zaidi et al., 2016). The CRISPR–Cas9 system using viral intergenic region (IR), CP, and Rep genes have been successfully used to impart resistance to BSCTV (Beet Severe Curly Top Virus) in transgenic *Nicotiana benthamiana* and *Arabidopsis thaliana* (Ji et al., 2015). However, Ali et al. (2015) showed the imposition of resistance to multiple geminiviruses viz. Tomato yellow leaf curl virus (TYLCV), Beet curly top virus (BCTV), and Merremia mosaic virus (MeMV) through CRISPR/Cas9 system in *N. benthamiana* by deploying a sgRNA aimed to recognize a conserved sequence (TAATATTAC) of IR (a characteristic of betasatellites).

Recently, CRISPR/Cas9-mediated genome editing tool was successfully used in cowpea (*V. unguiculata*) for the disruption of symbiotic nitrogen fixation gene by targeting symbiosis receptor-like kinase gene, which showed ~67% mutagenic efficiency as the complete blockage of nodule formation (Ji et al., 2019). Thus, the success of CRISPR/Cas9 in a *Vigna* system is expected to quickly promote functional genomics analyses for various other traits including YMD resistance in other *Vigna* species too. Like Cas9, Cpf1 is another type of CRISPR nuclease which is more efficient and result in lower off-target effect (Ji et al., 2019), and appears better alternative for the editing of various *Vigna* genomes, including mungbean for YMD resistance. Thus, CRISPR based genome editing approaches should be aimed to impose multiple virus resistance. Additionally, the CRISPR/Cas9 system may also be used for the identification of host factors controlling plant resistance through targeted mutagenesis (Zaidi et al., 2016).

## Conclusion and Future Thrust

**Figure 7:**
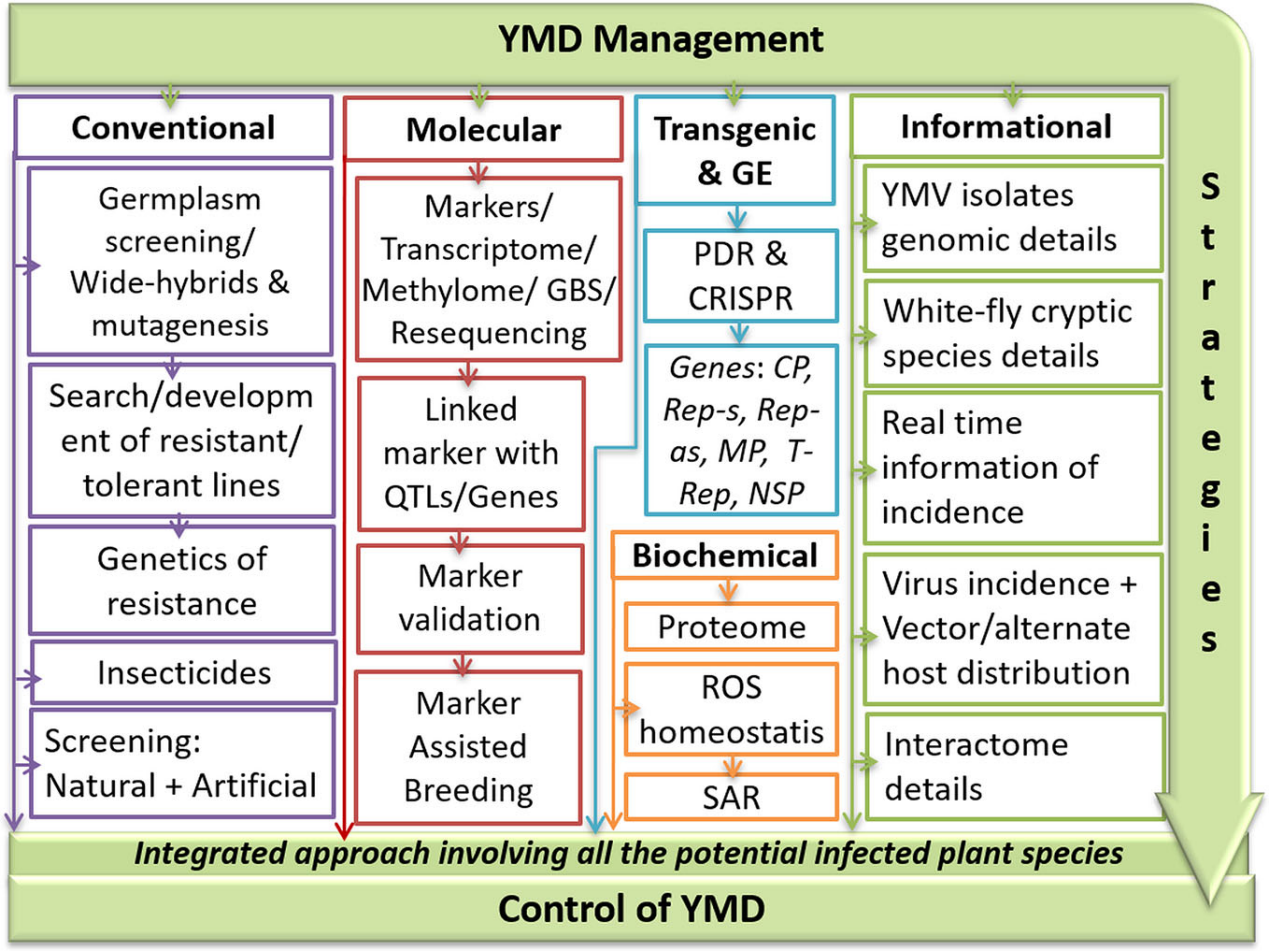
An outline of integrated YMD management strategies in mungbean. Where, CP, coat protein; CRISPR, clustered regularly interspaced short palindromic repeat; GBS, genotype by sequencing; GE, genome editing; MP, movement protein; NSP, nuclear shuttle protein; PDR, pathogen-derived resistance; QTL, quantitative trait loci; Rep, replication protein; ROS, reactive oxygen species; SAR, systemic acquired resistance; YMD, yellow mosaic disease; YMV, yellow mosaic virus.

Both mungbean crop diversity and MYMV affected area have gradually increased since the mid-nineties, which can be attributed to intensive mungbean farming. Any single YMD management strategy may not be a viable option as the resistance is governed by a range of factors like plant genotype, stains of YMVs, whitefly biotypes, ambient weather conditions, and presence of alternate hosts. Other YMD management challenges include (i) lack of precise infection mechanism of various YMV strains at the molecular level; (ii) development of multiple viral strain-specific resistant lines; (iii) reduction of vector population below threshold under field conditions. An inclusive outline of YMD development and management strategies are outlined in **Figure 7**.

The main reasons for not obtaining any durable resistance even after four-decades of YMD resistance breeding in mungbean could be due to the field-based germplasm screening without considering the natural existence of various begomoviruses along with the presence of whitefly cryptic species (Nair et al., 2017). Thus, any efficient YMD management strategy in *Vigna* should take into account the strains of YMVs, whitefly biotypes and their distribution in the target area (Nair et al., 2017) along with artificially screening through forced feeding and agroinoculation (Basak et al., 2005; Mohan et al., 2014).

The presence of various non-leguminous begomoviruses in legumes, suggests recombination in the virus, resulting in the appearance of more severe races, causing widespread crop loss (Ilyas et al., 2010). This again reiterates the pressing need for generating an exhaustive genomic database about the viral isolates affecting various crops across the world. The database should possess detailed phylogenetic information about MYMV and other isolates infecting different grain legumes. This will eventually facilitate in identifying the best strategy for the deployment of resistance sources having a mismatch of resistance gene(s) (Karthikeyan et al., 2014; Prema and Rangaswamy, 2018).

Comprehensive real-time information at the global level should be constantly generated in a network mode for the intensity of virus incidence and spatial distribution of vectors and alternate hosts for monitoring and giving early warnings about the possible occurrence of YMD (Meti et al., 2017). Such a system will also assist in making an appropriate judgment about the preventive and control measures, spray schedules, and other required practices for minimizing the YMD incidence.

The small genome size of the mungbean looks beneficial to the breeders for attempting genomic assisted breeding on a fast track for the development of YMD resistant varieties. The rapid advent of relatively low-cost RNA-seq technologies is also expected to assist in the mapping of the gene(s) or QTLs and MAS for YMD resistance. Although, various markers are reported closely linked with the YMD resistance gene, yet these are specific to some population and not yet validated across different sets of mungbean genotypes. Thus, a large number of linked SNP markers with the YMD resistance gene(s) should be identified with the aim of map-based cloning of the gene(s) (Maiti et al., 2011). Resequencing of different YMD resistant wild *Vigna* species from different geographical regions is expected to capture the allelic variations for the YMD resistance, whereas the use of advanced backcross-QTL (AB-QTL) may assist in the identification and transfer of valuable QTLs governing YMD resistance (Tanksley and Nelson, 1996). Detailed studies involving leaf proteome of different *Vigna* species may provide a deep insight into the YMD resistance response at the biochemical level. The flavin-containing monooxygenase identified through the association of proteomics data should be taken forward for overexpression analysis (Sai et al., 2017). Thus, information about the YMV infected host cell transcriptome, proteome, interactome, and degradome may give greater insight about the changes in the host cells and ultimately leading to the establishment of viral infection (Ramesh et al., 2017a). These -omics studies will also help in precise identification of various functional components which shows significant differential changes during both compatible and incompatible interactions.

The detailed information about the origin of dsRNA or the activation of plants RNA silencing machinery, when exposed to the YMV infection for imparting antiviral immunity in mungbean is still lacking. The *Ty*-1 and *Ty*-3 are the only host resistance genes identified for geminivirus infection in tomato, showing homology to host RNA-dependent RNA polymerases (Verlaan et al., 2013). This gave the clue about the role of secondary siRNAs as an effector in imparting RNA silencing-based antiviral resistance, but it warrants further evidentiary confirmation.

Due to its multiple host range, small genome size and larger carrying capacity, a geminivirus offers great prospects for its use in various novel applications including VIGS (virus-induced gene silencing) and genome modification involving ZFN (zinc-finger nucleases) (Kim et al., 1996), TALENs (transcription activator-like effector nucleases) (Miller et al., 2011), and CRISPR/Cas system (Zaidi et al., 2016). Thus, for sustainable YMD management, various novel and advanced biotechnological approaches especially gene editing, whole genome methylome studies, QTL-Seq, RNA-Seq and genome-wide association studies (GWAS) should be deployed for the inclusive understanding and management of YMD in mungbean.

## Author Contributions

GM, HD, RS, RN, SP: Conceptualization, writing and editing of manuscript. AK, AS, AR, KT, MA, NK, Priti, RK, UD: Collection and compilation of information.

## Funding

Financial support received from Indian Council of Agricultural Research (ICAR), New Delhi, and SERB, New Delhi (CRG/2019/002024) is gratefully acknowledged.

## Conflict of Interest

The authors declare that the research was conducted in the absence of any commercial or financial relationships that could be construed as a potential conflict of interest.
