# Corrigendum: Yellow Mosaic Disease (YMD) of Mungbean (*Vigna radiata* (L.) Wilczek): Current Status and Management Opportunities

**DOI:** 10.3389/fpls.2020.01064

**Published:** 2020-07-10

**Authors:** Gyan P. Mishra, Harsh K. Dikshit, Ramesh S. V., Kuldeep Tripathi, Ranjeet R. Kumar, Muraleedhar Aski, Akanksha Singh, Anirban Roy, Priti, Nikki Kumari, Uttarayan Dasgupta, Atul Kumar, Shelly Praveen, Ramakrishnan M. Nair

**Affiliations:** ^1^ Division of Genetics, ICAR-Indian Agricultural Research Institute, New Delhi, India; ^2^ Division of Physiology, Biochemistry and PHT, ICAR-Central Plantation Crops Research Institute, Kasaragod, India; ^3^ Germplasm Evaluation Division, ICAR-National Bureau of Plant Genetic Resources, New Delhi, India; ^4^ Division of Biochemistry, ICAR-Indian Agricultural Research Institute, New Delhi, India; ^5^ Division of Plant Pathology, ICAR-Indian Agricultural Research Institute, New Delhi, India; ^6^ Division of Seed Science and Technology, ICAR-Indian Agricultural Research Institute, New Delhi, India; ^7^ World Vegetable Center, South Asia, ICRISAT Campus, Patancheru, Hyderabad, India

**Keywords:** begomovirus, gene editing, greengram, pathogen derived resistance, translational genomics, vector management

## Error in Figure

In the original article, there was a mistake in **Figure 2** (Historical sketch of YMD in mungbean crop) as published. This figure has a picture of five Asian countries viz. India, Pakistan, Sri Lanka, Bangladesh, and Thailand, representing their political boundaries, which is erroneous. The corrected **Figure 2** appears below. 

The authors apologize for this error and state that this does not change the scientific conclusions of the article in any way. The original article has been updated.

**Figure 2:**
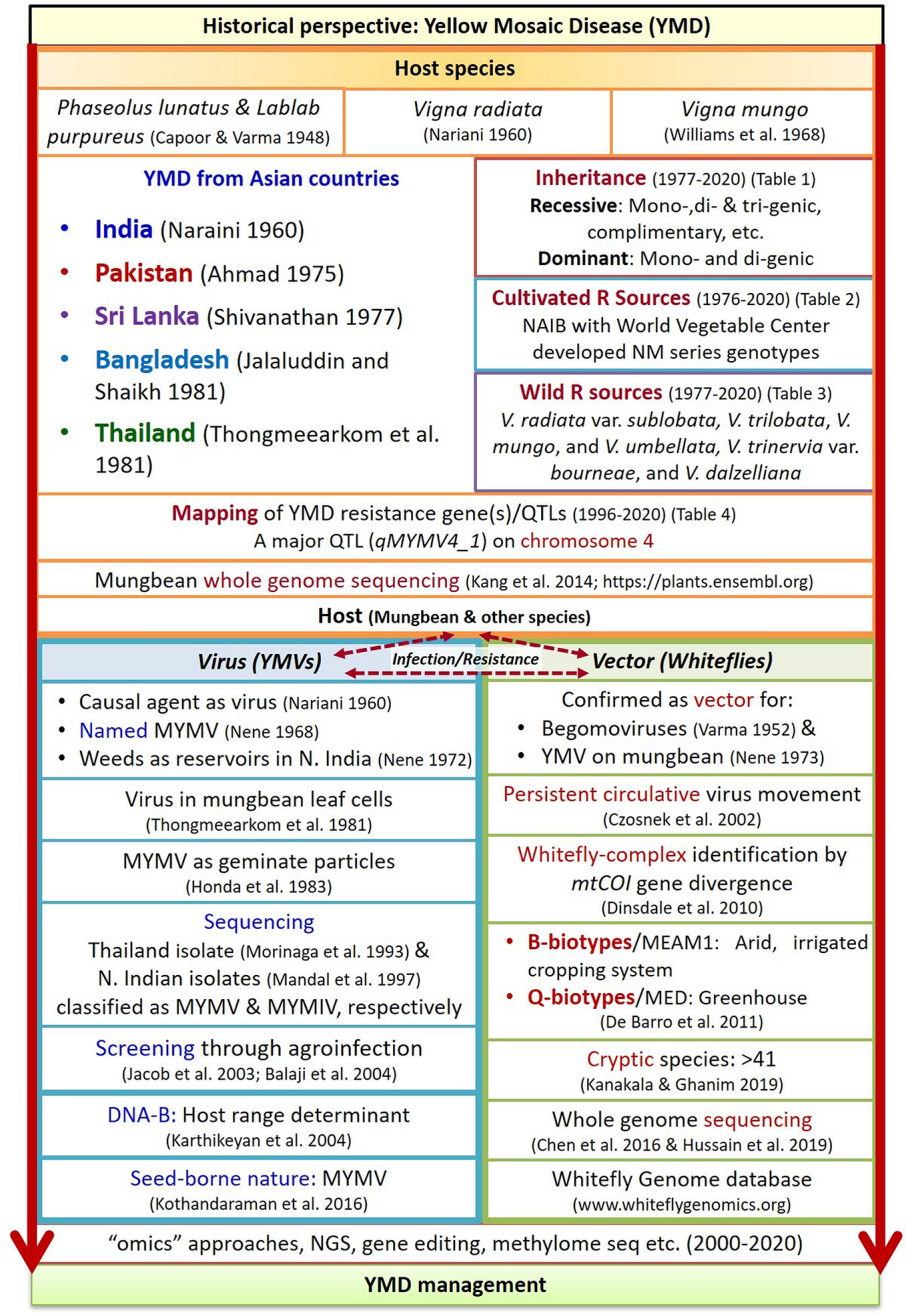
Historical sketch of YMD in mungbean crop (Derived from Capoor and Varma, 1948; Capoor and Varma, 1950; Varma, 1952; Nariani, 1960; Nene, 1968; Williams et al., 1968; Nene, 1972; Nene, 1973; Ahmad, 1975; Shivanathan, 1977; Jalaluddin and Shaikh, 1981; Thongmeearkom et al., 1981; Honda et al., 1983; Morinaga et al., 1993; Mandal et al., 1997; Czosnek et al., 2002; Jacob et al., 2003; Balaji et al., 2004; Karthikeyan et al., 2004; Dinsdale et al., 2010; De Barro et al., 2011; Kang et al., 2014; Chen et al., 2016; Kothandaraman et al., 2016; Hussain et al., 2019; Kanakala and Ghanim, 2019). Where, R, resistance; YMVs, yellow mosaic viruses; NGS, next generation sequencing.
