# Supplementary material for: Yellow Mosaic Disease (YMD) of Mungbean (Vigna radiata (L.) Wilczek): Current Status and Management Opportunities
Source: Front Plant Sci. 2020 Jun 24;11:918. doi: 10.3389/fpls.2020.00918 (PMC7327115; doi:10.3389/fpls.2020.00918)
Supplement: Supplementary file 1 [file Table_1.docx]

**Table S1. Primer details about the various MYMV components for viral DNA confirmation.**

| **S. No.** | **Primer sequence** | | **Remarks** | **Reference** |
| --- | --- | --- | --- | --- |
|  | **Fwd (5’-3’)** | **Rev (5’-3’)** |  |  |
|  | *FLDNAAF* (TGTG  GGA TCC ATT GTT GAA CGA CTT TCC C) | *FLDNAAR* (CAA TGG ATC CCA CAT TGT TAG TGG GTT CAG) | Full-length DNA-A of MYMIV | Hussain *et al*., 2004 |
|  | *BV1F* (CAA CAT CGA TAT GTT TAC TCG TAA TTA) | *BV1R* (CTT TAG TCG ACT TAT CCA ACG TAT TTC AAT T) | Nuclear shuttle protein (NSP) | -do- |
|  | *BC1F* (TTA TTA TCG ATA TGT TAC AAC ACT TTG TT) | *BC1R* (TTG GGT CGA CTT ATG ATT ATA ATT GTA AAC T) | Movement protein (MP) genes | -do- |
|  | *RepVI* (AAT GTA AAA GGC GAC TCA TA) | *RepCI* (GAG AAT TCA CCG GTC GCG GGG GCA) | MYMIV Rep in black gram | Biswas *et al*., 2009 |
|  | *MYMV-CP-F* (ATG GG (T/G) TCC GTT GTA TGC TTG) | *MYMV-CP-R* (GGC GTC ATT AGC ATA GGC AAT) | MYMV-Coat protein (1000 bp) | Naimuddin and Akram, 2010 |
|  | *MYMIV-DNA-A-F* (GTA AAG CTT ACA TCC TCC ACC AAG TGG) | *MYMIV-DNA-A-R* (TGT AAG CTT TAC GCA TAA TGC TCA ATA C) | MYMIV DNA-A conserved seq (~2.8 kb) | Ilyas *et al*., 2010 |
|  | *MYMIV-DNA-B-F* (CCA GGA TCC AAT GAT GCC TCT GGC A) | *MYMIV-DNA-B-R* (ATT GGA TCC TGG AGA TTC AAT ATC TC) | MYMIV DNA-B conserved seq (~2.8 kb) |  |
|  | *AV1P-F* (GTA TTT GCA (GT)CA (AT)GT TCA AGA) | *AV1P- R* (AGG (AGT)GT CAT TAG CTT AGC) | AV1P- MYMIV | Naimuddin *et al*., 2011b |
|  | *AC1P-F* (AGT TGA TAT GGA TGT AAT AGC) | *AC1P-R* (ACA AAA ACG ACT TCA AAT ATG CCA A) | AC1P- MYMIV |  |
|  | *AC2P-F* (AGC TAA TGA CCC CTA AAT TAT) | *AC2P-R* (GAG TAC TTG GAT GAA GAG AAC) | AC2P- MYMIV |  |
|  | *AC3P-F* (TTA TGA TTC GAT ATT GAA TTA ATA) | *AC3P-R* (CTG AAG TGT GGG TGT AGC TAT) | AC3P- MYMIV |  |
|  | *AC4P-F* (CAA ATT ACA ATT TAA GTT ATG) | *AC4P-R* (ACT TCT AGC CTT GTC AAC ACC AG) | AC4P- MYMIV |  |
|  | *MYMV-CP-F* (ACA CGA GCT CCT CTA CCC CGA TAT CGA ATG) | *MYMV-CP-R* (ACA CGG ATC CGT TGC ATA CAC AGG ATT TG) | MYMV-CP (~750 bp) | Islam *et al*., 2012 |
|  | *CPFP1-F* (GCG GAA TTA CGA TAC CGC C | *CPFP1-R* (GAT GCA TGA GTA CAT GCC) | Coat protein FP1 | Maheshwari *et al*. (2014) |
|  | *RT-CP-F* (GCG GAA TTA CGA TAC CGC C) | *RT-CP-R* (GAT GCA TGA GTA CAT GCC) | RT-PCR for CP (703 bp) | Sudha *et al*., 2015 |
|  | *Deng–F* (TAA TAT TAC C(GT) G(AT) (GT)G (AGC) CC(GC) C) | *Deng–R* (TGG AC(CT) TT(AG) CA(AT) GG(GCT) CCT TCA CA | Partial DNA-A in Black gram (530 bp) | Reddy *et al*, 2015 |
|  | *PAL1v1978* (GCA TCT GCA GGC CCA CAT YGT CTT YCC NGT) | *PAR 1c496* (AAT ACT GCA GGG CTT YCT RTA CAT RGG) | Partial DNA-A (1.5 kb) |  |
|  | *AV 494* (GCC (C/T)A T(G/A) TA(T/C) AG(A/G) AAG CC(A/C) AG) | *AC 1048* (GG(A/G) TT (A/G/T) GA(G/A) GCA TG (T/A/C) GTA CATG) | Partial DNA-A (500–600 bp) |  |
|  | *AC-abut* (GTA AAG CTT TAC GCA TAA TG) | *AV-abut* (AAA GCT TAC ATC CTC CAC) | Full length DNA-A (2.7 kb) |  |
|  | *BV-abut* (CCA GGA TCC AAT GAT GCC T) | *BC-abut* (ATT GGA TCC TGG AGA TTCA) | Full length DNA-B (2.7 kb) |  |
|  | *RHA-F* (TCA AGC TCC CGG TGC ATG TTG CA) | *AC-abut* (GTA AAG CTT TAC GCA TAA TG) | Right half of DNA-A (920 bp) |  |
|  | *DNA-B-F* (AGC CTA TGA CAC CGT CAA GAG GA) | *DNA-B-R* (CGC CGG GAC AAC GGC ATA T) | DNA-B (541bp) | Marabi *et al*., 2017 |
|  | *Rep-F* (GTC GAA TTG CTC GCT TCT T) | *Rep-R* (CCT AGA CTC GGT CGT TTT GC) | Replicase gene of MYMV | Sai *et al*., 2017 |
